# Supplementary material for: Designing and evaluation of the effect of community-based intervention on breast self-examination among reproductive-aged women in Ethiopia: A Cluster Randomized Controlled Trial
Source: PLoS One. 2025 Aug 7;20(8):e0329839. doi: 10.1371/journal.pone.0329839 (PMC12331034; doi:10.1371/journal.pone.0329839)
Supplement: S1 File — (DOCX) [file pone.0329839.s001.docx]

**Study Protocol**

**Design and Evaluation of the Effect of Community Based Intervention on Breast Cancer Screening Behavior among Reproductive Age Women in Ethiopia**

**BY**

1. **Feleke Doyore Agide (Ph.D., Associate Professor)**
2. **Gholamreza Garmaroudi (Ph.D., Professor)**
3. **Roya Sadeghi (Ph.D., Professor)**
4. **Elham Shakibazadeh (Ph.D., Professor)**
5. **Mehdi Yaseri** (**Ph.D., Professor**)
6. **Zewdie Birhanu (Ph.D., Professor)**

**Tehran, Iran**

**February, 2018**

# SUMMARY

**Background:** Breast cancer remains one of the deadliest non-communicable diseases in the world. Ethiopia is one of the countries with high breast cancer burden and faces the challenges from its devastating effects in terms of morbidity and mortality. According to the 2016 National Cancer Registry report of Ethiopia, it accounts for 33.4% of total cancer diagnoses in women. Therefore, the aim of this study is to design and evaluate community based intervention on breast cancer screening behavior among Ethiopian women.

**Methods and materials:** This study involves two phases. Phase 1 use qualitative method and phase 2 is a randomized controlled trial proceeded by cross sectional study lasting for six months will be conducted in Hadiya Zone, Ethiopia. Phase 1 is to design and develop the educational intervention. In phase 2, thirty clusters will be randomized by multistage cluster randomization technique, and allocated into two groups by permutation balanced block randomization. A total of 810 participants will be enrolled to the study by systematic random sampling technique. Educational intervention will be provided for the intervention group, while control group will be followed without any intervention. Open ended questions will be employed for qualitative part while structured questionnaire will be used for quantitative part. Content analysis will be used for qualitative data analysis. For quantitative analysis, multilevel logistic regression model will be used to consider for the intra-cluster correlation effect for quantitative analysis.

**Conclusion:** This will be the first study of its kind in Ethiopia to design and evaluate community based intervention on breast screening uptake. Therefore, it gives the opportunity to recognize the value of interventions and explore new intervention mechanism with an accurate method rather than the mere description of the problem. The unique identification number for the registry is **PACTR201802002902886.**

# ACKNOWLEDGEMENT

First and foremost, God deserves to take all the credits and best thanks in the name Jesus Christ for the inception and completion of this proposal work.

I am in doubt that this appreciation expresses their invaluable contributions; anyways, I take this golden opportunity to extend my boundless admiration and thanks to my supervisors and advisors for their priceless contributions and comments they have provided within the development of this proposal without which this proposal would not have appeared in this form.

I am glad to be in a position to thank Hadiya Zone health Department to their contribution in providing me baseline information.

Lastly but not least, I would like to express my deepest gratitude to Tehran University Medical Sciences for giving chance to conduct this research project.

# ABBREVIATIONS

**ANOVA**- Analysis of Variance

**BC**- Breast Cancer

**BCSU** – Breast Cancer Screening Uptake

**BSE** – Breast Self-Examination

**CONSORT-** Consolidated Standard for Reporting Trials

**CRCT**- Cluster Randomized Controlled Trial

**EDHS-** Ethiopian Demographic Health Survey

**EMA**- Expectation Maximization Algorithm

**EPHA**- Ethiopian Public Health Association

**EPHI**- Ethiopia Public Health Institute

**ETV**- Ethiopian Television

**FGD**- Focus Group Discussion

**GISA**- Global Strategies and International Affairs

**HBM**- Health Belief Model

**IDI** - In-depth Interview

**IEC**- Information Education Communication

**ITT**- Intention to Treat

**KAP** - Knowledge, Attitude and Practice

**MDG**- Millennium Development Goal

**RCT**- Randomized Controlled Trial

**RERB** - Research Ethical Review Board

**RERC**- Research Ethical Review Committee

**SNNPR**s- Southern Nation, Nationalities and Peoples Regional State

**SPSS**- Statistical Analysis Package for Social Sciences

**TUMS-IC**- Tehran University of Medical Sciences, International Campus

**US-** United States

**WHO –** World Health Organization

# TABLE OF CONTENTS

[Summary i](#_Toc506729580)

[Acknowledgement ii](#_Toc506729581)

[Abbreviations iii](#_Toc506729582)

[Table of Contents iv](#_Toc506729583)

[List of Figures vi](#_Toc506729584)

[Chapter One: Introduction 1](#_Toc506729585)

[1.1 Background Information 1](#_Toc506729586)

[1.2. Statement of the Problem 3](#_Toc506729587)

[1.3. Significance of the Study 5](#_Toc506729588)

[1.4. Objectives of the study 6](#_Toc506729589)

[1.4.1. General Objective 6](#_Toc506729590)

[1.4.2. Specific Objectives 6](#_Toc506729591)

[1.5. Research Questions and Hypothesis 6](#_Toc506729592)

[Chapter Two: Literature Review 8](#_Toc506729593)

[Chapter Three: Materials And Methods 18](#_Toc506729594)

[3.1. Study Area and Period 18](#_Toc506729595)

[3.2. Study Design 18](#_Toc506729596)

[3.3. Population 18](#_Toc506729597)

[3.4. Inclusion and Exclusion Criteria 19](#_Toc506729598)

[3.4.1 Inclusion criteria: 19](#_Toc506729599)

[3.4.2. Exclusion criteria: 19](#_Toc506729600)

[3.5. Sample Size and Sampling Technique 19](#_Toc506729601)

[3.5.1. Sample size determination 19](#_Toc506729602)

[3.5.2. Sampling techniques and procedure 20](#_Toc506729603)

[3.5.2. Recruitment plans 22](#_Toc506729604)

[3.5.4. Randomization 24](#_Toc506729605)

[3.6. Study Variables 24](#_Toc506729606)

[3.7. Data collection methods and procedure 26](#_Toc506729607)

[3.8. Data Quality Control 30](#_Toc506729608)

[3.9. Data Analysis 30](#_Toc506729609)

[3.10. Ethical Consideration 32](#_Toc506729610)

[3.11. Anticipated Limitations 32](#_Toc506729611)

[3.12. Strengths of this study 33](#_Toc506729612)

[Chapter Four: Plan of Activities (Work Plan) 34](#_Toc506729613)

[Chapter Five: Budget Break Down: 35](#_Toc506729614)

[References 37](#_Toc506729615)

[Annex I: Iformation Sheet And Consent Form 44](#_Toc506729616)

[Annex II: Questionnares 47](#_Toc506729617)

# LIST OF FIGURES

Figure 1.The conceptual framework of the model/research.……………………………….14

Figure 2. Schematic Representation of sampling procedure……………………………..20

Figure 3. Participants flow chart………………………………………………………….21

# CHAPTER ONE: INTRODUCTION

## 1.1 Background Information

Breast cancer (BC) remains one of the deadliest non-communicable diseases in the world. Ethiopia continued to be one of the countries with high breast cancer burden and faces the challenges from its devastating effects in terms of morbidity and mortality. According to the 2016, National Cancer Registry report of Ethiopia, it accounts for 33.4% of total cancer diagnoses in women [1, 2].

Worldwide strategies include health promotion and disease prevention approaches like early detection, breast self-examination, mammography use, breast clinical exam and early treatments [3]. It is recommended in low-income countries like Ethiopia, needs a similar approach that guarantees access to early detection with prompt treatment and prevention to all [4]. These strategies, while, simultaneously addressing the social and behavioral determinants of health in order to eliminate the catastrophic effects resulted from mortality of breast cancer [3, 4]. For the achievement of this strategy, intensified implementation research of innovations is one of the pillars [4].

Despite the endorsement and implementation of health extension program has shown a drastically improvement in disease prevention in Ethiopia; the burden of this disease in Ethiopia remains unacceptably high. The desired level of health seeking behavior for cancer screening has not been achieved as the WHO target and late stage reporting and severity is still a problem [2, 5].

Evidence from various research outputs indicate that outcome of breast cancer treatment largely depends on the timing of detection [5, 6]. Health promotion intervention is a key approach to increase uptake of breast cancer screening and early detection [6]. Widespread uses of community based health promotion and disease prevention strategies provide us with novel opportunities to advance early detection and treatment of the target groups [6, 7].

Therefore, the aim of this study is to design and evaluate community based health promotion interventions on breast cancer screening among Ethiopian women in the theoretical framework of the health belief model (HBM) in selected target group. The Model is more appropriate for assessing motivational variables, awareness and screening behavior of individuals in situations where the target groups have already in place where the services are there. Unlike other studies that go often unused for message design; the findings of this study will enable to design appropriate and effective intervention messages that will fit the audience specific need.

## 1.2. Statement of the Problem

Breast cancer is the most devastating public health problem affecting women all over the world. Worldwide, it is estimated that over 508,000 women died in 2011 due to breast cancer. Its incidence is increasing in the developing world due to increase life expectancy, urbanization and adoption of western lifestyles [8]. Although breast cancer is thought to be a disease of the developed world, almost 50% of cases and 58% of deaths occur in less developed countries [9].

Globally, the incidence and mortality rates of 43.1 and of 12.9 (per 100,000 ASR-W) respectively showing a five year prevalence of 239.9 [10]. In developing countries, where the incidence rate is expected to increase more, breast cancer gets high concern of public health importance [10, 11], while the highest incidence rates, shown in high-income countries, are partially to be attributed to earlier screening detection and treatment [12].

According to the American Cancer Society, approximately 230,480 females in the US were diagnosed with breast cancer [13]. The mortality rare in the same year was estimated at 39,520 [14, 15]. One in eight women born today will be diagnosed with breast cancer at some time in life [16].

In Ethiopia, cancer accounts for about 6% of total national mortality [17]. Although population-based data do not exist in the country except for Addis Ababa, it is estimated that the annual incidence of cancer is around 60,960 cases and the annual mortality is over 44,000 [2]. About 2/3 of annual cancer deaths occur among women [9]. Breast cancer takes highest percentage containing 33.4% of the total cancers [2, 17]. Ethiopian women typically present for care at a late stage in the disease, where treatment is most ineffective, and while system-related barriers to care account for a portion of that delay in access, women’s attitudes and lack of awareness of breast cancer symptoms account for a stalled initiation of action [2, 5, 18, 19].

A holistic approach which integrates prevention of modifiable risk factors for both breast cancer and other non-communicable diseases like promotion of healthy diet, physical activity and control of alcohol intake, overweight, obesity, could eventually have an impact in reducing the incidence of breast cancer in the long term [20, 21].

Screening is the most effective method to reduce morbidity and mortality from breast cancer. Breast self-examination, breast clinical exam and mammography are the method of choice for the early detection of breast cancer. However, its limited use in developing countries due to the high cost and limited availability make BSE a convenient and cost effective method, while less reliable [2, 14]. Many literatures confirmed the improvement in breast cancer outcome after self-examination, early detection, early diagnosis and mammography screening method [4, 22, 23].

The outcome of breast cancer treatment largely depends on the timing of detection. The health promotion interventions have immense contribution for early detection and improved survival. Indeed, studies have not been conducted or limited so far on the evaluation of perception of breast cancer screening uptake (BCSU) among reproductive age groups in Ethiopia.

Hence, findings from this study will provide a starting point for women to raise awareness amongst themselves, in the community as well as among their family about breast cancer and the importance of its breast cancer screening. Moreover, findings from the study can provide information on breast cancer screening for governmental health officials and other nongovernmental organizations which are working on breast cancer to raise awareness amongst women and the role of screening in breast cancer prevention and control program.

## 1.3. Significance of the Study

Successful behavior change intervention strategies require an in-depth understanding of factors that influence people to use the recommended responses/behavior. This study may help by providing assets that assist careful design & evaluation of preventive messages to reproductive age women via effective channels by pinpointing common factors that determine health seeking behavior of the women in general and breast cancer screening behavior in particular.

Furthermore, the findings of this study will enable policy makers, message developers, health educators, program designers and implementers, cancer prevention and control offices and used as baseline data/information to design appropriate and effective intervention messages that will fit the audience specific need.

| 1.4. Objectives of the study1.4.1. General Objective  - To design and evaluate the effect of community based intervention on breast cancer screening behavior among reproductive age women in Hadiya Zone, Ethiopia  1.4.2. Specific Objectives **Phase 1: Qualitative Objective**   - To explore factors to design and develop community based educational intervention on breast cancer screening behavior among reproductive age groups in Hadiya Zone, Ethiopia   **Phase 2: Evaluation objective**   - To assess perceptions towards breast cancer screening behavior among reproductive age women in Hadiya zone, Ethiopia - To evaluate the effect of community based educational intervention on breast cancer screening among reproductive age women in Hadiya Zone, Ethiopia. |
| --- |

### Research Questions and Hypothesis

- - 1. **Research Questions**
       1. What is the distribution of breast screening behavior in different predictor variables (socio-demographic, socio-economic, and perceptions)?
       2. Are the individual Health Belief Model constructs, confidence, health motivation, perceived susceptibility, perceived severity, perceived benefits, and perceived barriers, significant predictors of breast screening behavior?
       3. Does the community based health education intervention bring difference between intervention and controlled groups on breast screening behavior?
       4. Why women are not using breast cancer screening services or exploring barriers and facilitating factors for breast cancer screening behavior among reproductive age women?
    2. **Study Hypothesis**

1. There is a difference in breast screening behavior in different predictor variables (socio-demographic, socio-economic, perceptions) about breast cancer screening behavior among reproductive age women
2. There is a difference in breast screening behavior between intervention and control groups

# CHAPTER TWO: LITERATURE REVIEW

Health belief model based literatures have identified factors associated with uptake of breast screening in community where the service availability is ever growing and the emergence of the diseases need a rapid response. Naturally, the options are also available like breast self-examination, where service cost and limitation is null, and mammography and breast clinical exam. There are several theories and models that help to explain and predict health behaviors such as breast cancer screening uptake. The current study uses HBM as explanatory framework to guide review of the literatures.

**The Health Belief Model (HBM):** The Health Belief Model (HBM) is a socio-psychological model that attempts to explain and predict health behaviors in terms of certain belief patterns and by focusing on the attitudes and beliefs of individuals. It was developed by social psychologists in the 1950s in the United States Public Health Service to explain the lack of public participation in health screening and prevention programmes. Since then, it has been adapted to a variety of long and short-term health behaviors, including breast screening behaviors. The scholars of the HBM conducted major studies to explain preventive health behavior considering various perspectives such as health motivation and the individual's perceptions that can be influenced by prior experience as determinant to what an individual will and will not do [24].

The Health Belief Model (HBM) addresses the individual’s perceptions of the threat posed by a health problem (susceptibility, severity), the benefits of avoiding the threat, and factors influencing the decision to act (barriers, cues to action, and self-efficacy). It states specific health beliefs related with the health problem and recommended health actions influence likelihood of taking recommended health action (breast screening) [25, 26].

**The major constructs of the HBM and their definitions:** (a) Perceived Susceptibility: One's belief of the chances of getting a condition a health condition (breast cancer) (b) Perceived Severity: One's belief of how serious a condition and its consequences are or of leaving it untreated including evaluations of health and social threats. The combined effect of both perceived susceptibility to and severity of a health condition (breast cancer) result in perceived threat from ill health (severity of breast cancer) (c) Perceived Benefits: the subjective perception of the ease of the effectiveness of strategies designed to reduce the threat of illness. (d) Perceived Barriers: One's belief in the tangible and psychological costs of the advised behavior or subjective perception of perceived or real barrier of someone feels to take a recommended health action (breast screening). (e) Cues to Action: strategies to activate "readiness" to take a recommended health action (either physiological (e.g. swelling) or environmental (e.g., media peers) (f) Self-Efficacy: Confidence in one's ability to take action or one’s belief in being able to perform the recommended action [26].

In addition to the above basic constructs originators and researchers put some variables as modifying factors as they can influence individual perceptions and thus, indirectly health-related behaviors. These variables are of demographic, socio-psychological, health motivation and structural [25].

**Rationale and prediction in HBM**

The HBM is now used in explaining and predicting preventive health behavior, sick-role and illness behavior and has been applied to many studies of all types of health behaviors based on the understanding that a person will take a health-related action (undergo breast screening uptake) if that person:

| 1. | feels that a negative health condition [breast cancer] can be avoided/ progress can be reduced, | |
| --- | --- | --- |
| 2. | has a positive expectation that by taking a recommended action, she will avoid a negative health condition (i.e., getting screened will be effective in preventing breast cancer), and | |
| 3. | | Believes that she can successfully take a recommended health action (i.e., she can get screened and use health care services related to screening though barriers are there) [24, 25]. |

**Why health belief model in this study?**

This study uses HBM because in community levels after initiation of women are the decision makers as to whether they accept screening or not. Therefore, how women’s perceive the benefits of breast screening to one’s health and barriers of undergoing breast cancer screening may determine the decision in which part perception of threat of breast cancer. Thus, uptake of screening by women may be determined by perception of risk of breast cancer, health values, benefit of screening for one’s health and barriers which are perceived by women and socially constructed that HBM in this case relates beliefs about breast cancer with its screening [24].

**Strength, modification and conjectures while using HBM in this study**

At first, to overcome limitations of the model, a) the constructs of health belief model were considered in a way that social factors related to uptake of screening will be contextually addressed b) breast cancer screening and access to related services are given on central part of the country that reduce the problem of enabling factors like economic factors. Secondly, HBM is a model that associates a certain health problem with its levels of prevention, of behavior change models, while the rest more or less just deal with preventive behaviors needed. Thirdly, to increase the variance HBM explains in community and service providers setting additional constructs were incorporated as effect modifiers of main constructs and to help explain more the prediction of uptake of screening particularly. These constructs were; a) general health motivation b) past behavior related to screening and c) distal factors Therefore, in this study HBM predicts uptake of screening through relationship between knowledge of breast cancer, risk behaviors, women’s perception of risk, severity of breast cancer, the benefit and barriers of screening, health motivation and self-efficacy, prior experience of screening, and perception irrationally expressed (distal factors) and initiation. Accordingly, literatures that showed determinants of uptake of screening in various settings including community setting will be reviewed based on concepts and constructs of the modified Health Belief Model.

**Individual perceptions of breast cancer**

**Perceived susceptibility to breast cancer**

A study in USA and Paris reported weak evidence that personalized risk communication increases uptake of screening tests (OR 1.5, 95%CI 1.11 to 2.03 and OR 1.31; 95%CI 0.98 to 1.77) with a smaller effect for mammography. In the same study, whether numerical calculations of risk are used and presented, the OR for test uptake was 0.82 (95% CI 0.65 to 1.03). In the same study, the risk estimates or calculations, which were categorized into high, medium or low strata of risk, perceived susceptibility takes a highest place [27].

Systematic reviews conducted in the United States, Asian women, Hispanic women and other western countries on breast cancer screening uptake showed that factors associated with uptake of screening rates included the client's perception of breast cancer risk, acknowledging risk behaviors, confidentiality protections, considering screening uptake as 'routine' rather than optional and the health workers/professionals belief that screening will benefit the woman and the factors associated with low acceptance rates included prior experience of the screening, fears about coping with results, and explicit informed consent [20, 28-31]. A study conducted in Ethiopia in Kaffa Zone revealed that breast self-examination performance among female teachers was very low [5].

**Perceived severity of breast cancer, perceived threat from breast cancer and cues to action**

A various studies the financial impact of breast cancer can be quite significant, and studies have shown that women diagnosed with the disease are especially likely to experience financial hardships [32-35].

Next to cardiovascular disease, cancer is the second most expensive disease in the United States, with an estimated annual cost of $171 billion [36], and specifically, annual breast cancer-related medical treatment costs, specifically, are now estimated at $8.1 billion [37, 38]. Physiological and psychological issues compound the financial impact of breast cancer [39]. For example, certain treatment methods (such as surgical removal of the breast, or mastectomy) can result in permanent disfigurement and subsequent tingling sensations, numbness, and chronic pain [40].

In addition to the physiological impact of breast cancer, women are also likely to experience psychological and social consequences related to the disease like depression, worry, anxiety, and isolation are among the various emotions women may feel at any given point in her experience with breast cancer [41]. Moreover, many women deal with issues related to body image because they oftentimes find that breast cancer screening changed the way they looked; such changes include hair loss, skin-related issues, and weight gain or loss [41, 42].

In Ethiopia in 2010, the overall death rate due to breast cancer was roughly 6% and more than 95% is females [16, 43, 44]. In addition, as recently as 2016, an estimated 60,960 new cases of breast cancer in women were diagnosed in this central part of the country and annual mortality is 44,000 [45, 46]. Further, even though mammography screening has been shown to reduce mortality due to breast cancer by approximately 11% over 10 years among women aged 30-49 years and older, in 2010, 31.4% of women in Ethiopia in the 40-plus age group identified not having had a mammogram within the last two year [47].

**Perceived benefits of breast screening**

A randomized controlled trial on various parts of the world on breast screening shows among those who had not screened, none of the breast cancer risk factors examined (breast mass, inflammations) was associated with intention to be screened for breast cancer, after taking other factors into account [48-51]. Those who perceived the benefits of screening as it helps a person to live longer and talked to others about breast cancer were more likely to intend to be screened [51, 52]. The existence of mammography, the awareness of self-examination and the availability of breast clinical services were perceived as the most important factors [52].

Making cancer screening information and services available to women is essential for reducing high rates of cancer and cancer deaths [49, 52]. Early detection can reduce mortality significantly and will promote women’s overall quality of life [13, 49, 53, 54].

**Perceived barrier of breast screening and Self-efficacy**

In a systematic review and meta-analysis conducted up to 2015 on the effectiveness interventions on breast screening in Asia and USA, health care workers into value their health. The perception of how confidentiality is handled influence women’s willingness to be screened [20, 21].

A study conducted in South Africa in uptake of screening among women after offering education shows uptake rate was increased [55, 56]. In another study, in the same country, the reasons for not screening were having already been screened for breast cancer, felt unready to be screen, the need to consult with partner, and refusing with no explanation [56-58]

In a cross sectional study conducted on attitude and perception on breast cancer screening in Ethiopia in 2011, experience among those who had been previously screened suffered from signs and symptoms, discrimination, and gap of healthcare worker confidentiality [59].

A study conducted on factors affecting cancer survival and screening among women in southern Ethiopia in 2014 shows women’s education, residing in urban, having open discussion about breast cancer screening with partners were statistically associated with screening uptake while among those who were not willing to be screened stigma and discrimination by the community were reasons for refusal of the screening [60].

**Modifying factors**

**Past behavior related to breast screening and experience of screening**

A randomized controlled study conducted in USA on routine breast screening in migrant women shows; more than half agreed to breast screening, with no new symptoms detected [49]. Common reasons for not screening were perceived low risk and self-reported breast screening previously and being younger age, Hispanic ethnicity and having another screening during the visit were independently associated with breast screening [61].

A study conducted in the United States on evaluation of acceptability of breast cancer screening shows acceptance was generally higher among women at high risk for acquiring than among low-risk persons [49, 61]^.^

A cross sectional study conducted on behavioral survey for breast screening in Addis Ababa among the general population those who didn’t have adequate knowledge of breast cancer, considered themselves at low risk [62]. In another cross sectional study, the findings revealed that only 156 (57.8%) of them were knowledgeable about breast cancer and its screening and 114 (42.2%) were not [59].

According to Ethiopian cancer (oncology) report in 2016, about 33.4% out of the total is breast cancer which accounts 6% of all deaths in Ethiopia [59, 63]. The perception they have in positive or negative way leads women to consider oneself to be at medium or high chance of infection were looking breast mass, breast inflammation, experience from others, being female and unrecognized pain in breast, lack of awareness and feeling of health and economic issue and negligence [63].

**2. Socio-psychological factors [Perception of impossibility to screening]**

In a survey in general population based in Ethiopia regarding routine screening while the majority of respondents reported that routine screening was beneficial [2, 64]. Given the high social status of health professionals, the shortage of healthcare services (mammography) and the arguably universal psychological tendency to obey authority, women may be unlikely to oppose the recommendation of physicians and healthcare institutions [2, 65].

**3. Socio demographic characteristics and knowledge of breast cancer and its screening**

A community based study in 2005 in USA on intervention to increase screening mammography among women 65 and older shows adjusted correlates of breast screening included higher education, perceived access to screening services, fear of result, and fear of discrimination positively correlated toward breast screening [66].

A Cross sectional study conducted in 2011 on assessment of Knowledge of Breast Cancer and Screening Methods among nurses university hospitals in north Ethiopia showed that knowledge on breast cancer, ever heard of breast screening and its services, attending formal education, high awareness about the benefits of screening, low stigmatized attitude were strongly associated with uptake of breast screening [59]. In the same study and other breast self-examination studies, unemployment and self-perceived high risk of breast cancer were associated with initial willingness. However, only being unemployed and house wife was associated with not to be screened [59, 67, 68].

With reference to the review, literatures showed research on breast screening uptake should move from the problem definition stage to the next phase of assessing the value of interventions among women. And also it has a clear indication of further research is required to develop new simple, brief, and effective interventions to boost breast screening uptake. Thus, taking these and other questions in to account which has to be answered, this study will conducted on the effect of community based health promotion intervention on breast cancer screening uptake among reproductive age women of Ethiopia using modified health belief model.

**BC ill health perception Modifying factors Likelihood of BC screening uptake**

**- Perceived benefits from breast screening**

**- Perceived barrier to breast screening**

**- Sociodemographic characteristics**

**- Socio-economic characteristics**

**- Socio-psychological characteristics**

**- Knowledge about breast cancer and breast cancer screening**

**Health motivation**

Perceived susceptibility to breast cancer

Perceived severity of breast cancer

Likelihood of performing behavior

(Uptake of screening)

**Perceived threat of Breast cancer**

**Cues to breast cancer screening**

Self-efficacy to undergo breast screening and use related services

**- Past behavior related to breast cancer and breast screening**

**- Distal factors**

# Fig 1: Conceptual framework of the research

# CHAPTER THREE: MATERIALS AND METHODS

## 3.1. Study Area and Period

This study will be conducted in reproductive age women of Hadiya Zone in southern Ethiopia. Hadiya zone is among the thirteen zone southern nations, nationalities and peoples of Ethiopia. The zone has ten woredas and two city administrations and 332 kebeles and “kifleketemas”. Its capital city is Hossana which located 230 km South East of capital city Addis Ababa and 194 km form the regional city Hawassa. The estimated population of the Zone is 1,650,104. The male population is 817,626, and the estimate of females in child bearing age (15-49) is 193,967. In the zone, in each kebele, there is at least one health center and health post. The total number of health institution by type is corresponding to kebele numbers and more. The communicable and some non-communicable diseases prevention work is being done by health extension workers and community health agents. Study period will be conducted from March to November 2018.

## 3.2. Study Design

A cluster randomized controlled trial proceeded by cross sectional study lasting for six months will be used to evaluate effectiveness of the community based educational intervention among women of reproductive age groups in Hadiya zone in southern Ethiopia. In fact, in first phase, qualitative method is employed for designing and developing the intervention.

## 3.3. Population

**For quantitative part,**

**3.3.1.** The source population will be women of reproductive age (15-49) who are living in

Hadiya Zone during the study period.

**3.3.2.** The study population will be women of reproductive age (15-49) who will be sampled

from the source population and fulfilled inclusion criteria.

**For qualitative part**, in phase 1, focus group discussions and in-depth interviews will be conducted with women and health workers (health extension workers, medical directors, and disease prevention and health promotion officers).

## 3.4. Inclusion and Exclusion Criteria

### 3.4.1 Inclusion criteria:

1. Women in reproductive age group
2. Women who are physically, mentally capable to provide informed consent, and can follow intervention provided without any support
3. Willing to participate and give data to the researcher.

### Exclusion criteria:

1. Participant who has already attended another intervention program.
2. Participants who cannot stay until the intervention is completed/moveable participants during intervention time

## 3.5. Sample Size and Sampling Technique

### 3.5.1. Sample size determination

For cluster level intervention, the sample size is calculated using double population proportion formula for cluster randomized trial [76, 77], with the assumption of the percentage of screening rate (π_0_) at the base line is similar in both groups; and Z_α/2_ the standard normal variable, at α = 5%; Z_β_ is (1-β) = 80%; **π_0_** is the prevalence of the participants who have knowledge about breast self-examination as screening methods since the affordable behavior is BSE (77.6%) [78]; a variable which gives maximum sample size; **π_1_** is the prevalence of screening rate in the intervention cluster/ kebele (87.6%) (Assumed to be increased by 10%); k is coefficient of variation of true proportions of the outcome variable between the clusters within each group. Since there is no study to estimate k, it is taken as 0.25.

**(Zα/2+Zβ)^2^ X [**π**_0_ (1-**π**_0_) +** π**_1_(1-**π**_1_)]**

**n = ___________________________________**

**(**π**_0_-** π**_1_)^2^**

Then, the sample size will be:

**(1.96+0.80)^2^ X [0.79(1-0.776) + 0.876(1-0.876)]**

**n = _________________________________________**

**[0.876-0.776]^2^**

**n**= **245** for each groups

Because, our study is cluster randomized trial, we assumed design effect 1.5 by cluster sampling method to overcome the design effect. Then, the sample size will be:

**245*1.5 = 367.5 ≈ 368**

Finally, sample size further increased by 10% to account for contingencies such as non-response or recording error, i.e. 368 X 10/100 + 368= 404.8 ≈ **405**. Therefore, the final sample size will be 405 women for each group. Hence, the total number of study clusters/kebeles will be 30 i.e.15 kebeles in each arm based on WHO cluster sampling recommendation and then proportionally distributed according the number population in the cluster [69].

### Sampling techniques and procedure

Hadiya zone has twelve districts (ten woredas and two city administrations). For sampling, in phase 1, ideally sample size for qualitative research depends on data saturation. For now, six focus group discussions proposed to be conducted with women and nine in-depth interviews will be conducted with health workers in selected six districts. Phase 2, we will select six districts randomly by multistage cluster random sampling method. Then, we will select 30 kebeles from the selected districts and we will evenly distribute in intervention and control groups (i.e. 30 kebeles will be divided into 15 control and 15 intervention groups). To prevent information contamination, random assignment will be used to make control and interventional sites are far from each other. As shown in figure 2 below, we will divide 810 participants proportionally for each selected clusters/kebeles. Hence, the total number of study clusters will be 30 i.e.15 clusters/kebeles in each arm based on WHO cluster sampling recommendation [69].

**Twelve districts (Ten Woredas and two city administration in Hadiya Zone, Southern Ethiopia)**

**Six districts (Four woredas and two city administration (30 kebeles) = 810 women participants)**

**0**

**Three districts (Two Woredas and one city administration in control arm (15 kebeles))**

**Three districts (Two districts and one city administration in intervention arm (15 kebeles))**

**405 samples for control arm from each district accordingly**

**405 samples for intervention arm from each district accordingly**

# Fig 2. Schematic presentation of sampling procedure

### 3.5.2. Recruitment plans

For the participant enrollment, in phase 1 qualitative data will be collected from women, heath extension workers, medical directors and disease prevention and control officers. In phase 2, women who are potentially eligible for the study will be listed, and each individual will be invited to participate in the study through oral invitation or phone call performed by principal investigator and health extension workers of each selected kebeles/clusters. An appointment will be made if the person agrees to participate. Following an oral informed consent, women will be included for eligibility as subjects of the study by a research member, baseline data will be obtained, and participants will be allocated either intervention or control group. Written informed consent will be obtained from the women who are willing to participate in the study.

ITT **and** Effectiveness analysis

**Randomization of Participants (n= 810)**

**Informed Consent and**

**Baseline Data Collection**

**Participants’ Recruitment Procedure**

**Design and development of intervention**

**Inclusion Criteria:**

-Women in reproductive age group

-Women who are physically, mentally capable to provide informed consent and can follow intervention provided without any support

-Willing to participate and give data to the researcher.

**Exclusion criteria:**

- Participant who has already attended another intervention program.

- Participants who cannot stay until the intervention is completed/ moveable participants during intervention time

**Intervention group (n= 405)**

- Welcome message,
- Health education
- Brochures and posters
- Perception assessment and positive feedback
- Counseling healthy behavior plan
- continuous follow up

**Control group (n=405)**

- Usual care (services)
- Welcome message
- No continuous follow up

Phase 2 follow up assessment at the end of 3^rd^ and 6^th^ month

**Main outcome:** Screening Uptake **Secondary outcomes:** Increased knowledge, attitude and intention to screening

# Fig. 3 Participants Flow Chart

- - 1. **Method of assignment to study groups**

### Randomization

Randomization will be carried out using a computer program by the investigators. The different kebeles will be alphabetically coded (A, B, C, D and so on) and participants attached to each kebele will be given numerical codes (for example, participants in kebele Y will be numbered Y001, Y002, Y003 and so on. Participants in the randomized controlled trial will be assigned 1:1 to the intervention and control arms under restricted randomized design after informed consent and collection of baseline data. The allocation sequence will be generated and released to the interventionist on a case-by-case basis by another independent department specializes in generating research random sequence. Interventionist, data collectors, statistician are not the same persons. Interventionists also acknowledge all those contacted are in the intervention arm. But anonymous responses will be entered into the database by a person unconnected with the project.

## 3.6. Study Variables

**3.6.1. Dependent variables**

- Breast screening behavior/ uptake (breast self-examination, mammography use, clinical exam)
  - 1. **Predictor variables: (modifying factors, experience and perceptions)**
- **Socio demographic**, socio-psychological and structural characteristics ( age, marital status, religion etc., general knowledge about breast cancer and screening, health motivation/values)
- **Socio-economic variable:** income, occupational status, wealth index
- **Past behaviors related to breast cancer and experience of screening (**knowing status of partner, screening experience).
- **Perceived susceptibility** to breast cancer
- **Perceived severity** of breast cancer
- **Perceived benefit (response efficacy)** of screening in relation to attitude towards cancer treatment, belief for treatment
- **Perceived barrier** of uptake of screening ( fear of being cancer positive, fear of discrimination and stigma, perception of confidentiality, previously knowing the provider, time to think over for readiness, perceived knowledge about the service, perception about non acceptance of accompany)
- **Self-efficacy** to have screening and to live with breast cancer.
- **Cues to actions:** Facilitating and triggering a women to uptake of screening
- **Distal variables** (factors emerged from qualitative parts)

**Constructs for qualitative study:**

- Facilitating for and barriers of screening
- Existing and cultural knowledge for breast cancer and its screening
- Nature and reason of uptake of screening
- Women’s rational decision in undergoing breast screening.
- Irrational ways or defense mechanisms/perceived
- Worrying issues

**3.6.3. Outcome measures:**

Outcomes will be compared between intervention and control arms, and between different time points of each arm. The primary outcomes include breast cancer screening behavior/intention (mammography use, breast self-examination and breast clinical exam), secondary outcomes include increased awareness for breast cancer and its screening and developing health motivation. Another outcome is, not a must, community based intervention mechanism using HBM constructs effectiveness. The outcome for qualitative part will be measured with respect to constructs of health belief model and anomalies treated accordingly. If anomalies are there, they will be used in questionnaire amendments to be included in quantitative and treated as distal factors in the conceptual framework.

## Data collection methods and procedure

Both qualitative and quantitative methods of data collection will be considered in this study. In phase 1, qualitative study will be conducted for development and design of intervention. In phase 2, baseline data of all the subjects will be collected at the enrollment procedure. The measures including demographic information (age, marital status, current occupation, educational level, income and previous residence), knowledge about and perception of breast cancer and screening, past behavior related to breast cancer screening and distal factors. At the end of the intervention (after three months), the same data will be collected. To see the effect of the intervention, the same data will be collected at the end of the six months.

**3.7.1 Data collection instruments:**

For qualitative part of the study (phase 1 for designing intervention), focus group discussion and in-depth interview guide comprising: women, health providers and health extension workers experience regarding breast cancer, and barriers to and facilitating factors for breast cancer screening. Perception of women’s rational decision/based on critical thinking in breast screening uptake or not

In phase 2, a pretested, structured and translated questionnaire adapted from various breast cancer screening related studies [49, 61, 70-75], based on modified constructs of health belief model will be used as instrument for quantitative study. The translation will be made from English language to Amharic and back translated to English version by different individuals who are blind to the original version of the questionnaires (English version) in order to facilitate reliable responses to underlying questions and keep the original meaning of the instrument. The instrument comprised socio-demographics characteristics, knowledge about breast cancer and screening items with response format of ‘yes’, ‘no’ and ‘don’t know’ assuming score of ‘Yes’=1, ‘No’ or either of ‘don’t know’=0 for every correct item and will be reversed for incorrect items, Cues to breast screening with dichotomized ‘yes’, ‘no’ items and past behavior related to breast cancer and experience of screening items with a mix of nominal and scale measurements. Womens’ health value and Health motivation, perception of susceptibility to and severity of breast cancer, perceived benefit breast screening, perceived barrier of screening and self-efficacy to take screening and related health care items all of which eliciting responses on a five-point Likert scale format, ranging from `strongly disagree' to `strongly agree' will be incorporated. Each of the responses will be scored as: `strongly disagree' = 1, `disagree' = 2, `undecided/not sure' = 3, `agree' = 4 and `strongly agree' = 5. After reversing for negatively worded items, scores will be summed for each respective concept.

Factor analysis will be done for validation of the instrument. For this, Eigen value of greater than one will be considered for construct validity and confirming constructs of the model. Factor loading score of ≥ 40% and rotation with varimax method will be considered to identify to which construct each item belongs that items with factor loading score of less than 40% will be rejected. After constructs are decided on those constructs previously lacking names will be named according to the concept the loaded items inferred of the constructs. To ensure reliability of the scales, internal consistency of items will be seen separately for each construct identified using cronbanch’s alpha score of ≥ 70% as cut of point. Items correlation with total correlation of score ≥ 30% will be acceptable cutoff point and less will be avoided.

**3.7.2. Procedures**

**3.7.2.1. Phase 1: Qualitative data for designing and development of community based intervention**

For this, first, focus group discussion with women and in-depth interview with health promotion and disease prevention experts, medical directors and health extension workers will be conducted by using a semi-structured checklists, which involves questions about currently challenging behavioral, cultural and social problems, facilitating factors and barrier of uptake of breast screening, ever used or currently predicted health promotion intervention applications and their characteristics, preferences of name and functional needs about a breast cancer screening message especially for women. Ideally, sample size of the qualitative will depend on the information saturation rule which means as sample expanding, no new theme be induced from information the participants supplied. A community based intervention will be prepared based on the results. Second, the research team will discuss on the issue and design the intervention considering feasibility, applicability and acceptability and expert experiences for the message delivering. Finally, community based intervention document will be determined and prepared. After the document is completed, we will invite the research team again to test it, necessary modification will be made accordingly.

**3.7.2.2. Phase 2: Evaluation of Effectiveness of Community Intervention (Intervention description)**

Participants in the intervention arm will receive community based educational intervention. Each of them will be required to follow the educational intervention which will be given every 15 days for 3 months and should register their names and phone numbers (even family phone numbers) for tracking and reminding purpose. To do this, we, research team, will take the following points into consideration: first, building rapport (good interaction); second, facilitating supportive environment including social and psychological environment; third, motivation of participants to use the screening services and encouraging the positive act. Finally, women centered empowerment words and languages to maintain the health issue to be sustained in the groups. This makes not to be snap shot action for the participants to value their health in future endeavors. All of the participants will be promised of the confidentiality throughout the process.

**3.7. 2.3. Control group**

The control group will receive usual services from health extension workers. These participants will only receive a welcome message at the beginning, to validate their entry into the study, and a message at the end of the follow up to thank them for their participation. These participants will not receive specially prepared community based educational intervention.

**3.7.2.4. Participant follow-up**

All of the participants in control and intervention arms will attend the three visits, one at baseline after three months, and the other at the end (after six months).

**3.7.2.5. Intervention**

Educational intervention will be prepared based on health belief model constructs and distal factors which are interlinked with breast screening behavior. In addition, the intervention will be emerged from qualitative parts will be treated as distal factors in the model and used as very important base for intervention designing. Educational intervention will be provided on mammography, breast self-examination and breast clinical exam by training and teaching using different methods and materials like posters and brochures.

**3.7.3. Data Collectors**

In both phases, health professionals who hold B.Sc. degree and above will be recruited for data collection of qualitative, baseline, after three months and end of intervention (after six months). In case of qualitative, principal investigator act as data collector together with other data collectors. The same sex moderator will be used to reduce sensitivity. Supervisors who hold B.Sc. degree and above will also be assigned for supervision.

## Data Quality Control

As it has been explained above in the sampling part, districts (woredas/city administrations) for control and interventional groups will be selected randomly while considering the optimum distance between control and intervention group to prevent information contamination and to distribute socioeconomic variables difference between districts. The instrument designed based on literature review will be approved for validity and reliability. Community based intervention based on health belief model constructs will be designed by the research team who are in the field health education and promotion and applied thoroughly by trained health professionals. Data collection will be supervised and checked before electronic data entry by principal investigator and other study team member closely supervise to assure the maximum quality level. Then, double data entry verification method will be employed in separated spread sheet and data accessibility will be restricted to data manager and study principal investigator. Before analysis, data will be checked for normality and homogeneity, then analyzed & interpreted by research team and biostatistician. Finally, quality scientific report will be developed and disseminated.

## Data Analysis

Qualitative data will be analyzed by Atlas ti.7 software and use narration and direct quotation. For quantitative, analysis at baseline**, e**quivalence of two arms will be analyzed at baseline. The equivalence will be analyzed by using Mann- Whitley U test, Chi-square test, T-test or ANOVA.

**Analysis of intervention outcomes:** Consolidated standards of reporting trials (CONSORT standards) will be followed for reporting in this study [79]. For intervention arm and control arm comparison, indicators of rates of breast screening, breast self-examination and/or breast clinical exam at baseline, after three months and at the end of six months will be compared by using Chi-square and T-test. And logistic regression model will be used for predicting independent predictors of breast cancer screening behavior.

**Intention-to-treat (ITT):** Outcome will be analyzed under the basis of ITT. First, the principles of ITT will be applied to test the effects of attrition [80], which means that all participants who have been randomized will be included in the analysis. Missing endpoints will be imputed by using the Expectation- Maximization (EM) algorithm. To gauge the robustness of the outcomes, this analysis will be repeated while using Multiple-Imputation approach. Second, propensity score analysis will also be used. By using logistic model (1 for screening uptake and 0 for not) to predict the probability of be in breast screening behavior. And then use the predicted variable to replace the attrition of dummy variable.

Analysis of intervention mechanism in correlation analyses between each aspect of participants’ community based intervention adoption/use behaviors (e.g. frequency of breast screening by mammography, breast self-examination and/or clinical exam) and effectiveness of the intervention will be performed. A causal path model will be built according to correlation analyses results and related theories. And a path analysis will be subsequently conducted so as to calculate the residual path coefficient and determination coefficient, define causal relationships between different variables, and thus explore intervention mechanism of the community based intervention.

## Ethical Consideration

This study will strictly follow the ethical principles of the Helsinki declaration of medical research [81]. Prior to starting any research process, ethical approval will be obtained from the research ethical review and approval board (RERB) of Tehran University of Medical Sciences, International Campus (TUMS-IC), and the Research and ethical review approval committee (RECC) of Ethiopian Public Health Institute (EPHI). The letters written from TUMS-IC and EPHI will be given for Hadiya Zone health administration department for legal permission. Both oral and written informed consent will be obtained from each participant after thoroughly explaining objectives and benefits of the study. To ensure confidentiality, any personal information on participants will not be collected and psydeo-names will be given during discussion of study. Data collected for this study will not be used for other study without approval of each participant. At the end of the data collection for evaluation part, the same education will be provided for controlled groups. All international and institutional randomized control trial research ethics conventions will be strictly obeyed during our study process. This study has been registered in Pan African Clinical Trial Registry (www.pactr.org) database with unique identification number for the registry is **PACTR201802002902886.**

## Anticipated Limitations

- One limitation of this study is that the control group received a usual intervention which may confound the intervention given in intervention arm. Health extension workers may be motivated more than usual time to teach them after the start of this research.
- Another limitation of this study is that the study used health promotion intervention at community level which by far used to reach unreached community, in case information contamination may exist due to the nature health promotion research.

## 3.12. Strengths of this study

- This is one of the first cluster randomized controlled trials in Ethiopia to implement community based intervention beyond conventional observational studies particularly cross-sectional study.
- The other strength of the study is the research team used qualitative method and other relevant reviews before designing and development of intervention. The intuitively known scholars are participated in this research through development of intervention mapping and modules.
- This will be the first study of its kind in Ethiopia to design and evaluate community based intervention on breast screening uptake. It may help the researchers to recognize the value of interventions and explore new intervention mechanism with an accurate method rather than the mere description of the problem.

# REFERENCES

1. Tigeneh, W., et al., Pattern of cancer in Tikur Anbessa specialized hospital oncology center in Ethiopia from 1998 to 2010. Int J Cancer Res Mol Mech, 2015. 1(1).
2. Abate, S., et al., Trends of breast cancer in Ethiopia. Int J Cancer Res Mol Mech, 2016. 2(1).
3. Sankaranarayanan, R., et al., Clinical breast examination: preliminary results from a cluster randomized controlled trial in India. Journal of the National Cancer Institute, 2011. 103(19): p. 1476-1480.
4. Yip, C.-H., et al., Breast cancer management in middle-resource countries (MRCs): consensus statement from the Breast Health Global Initiative. The Breast, 2011. 20: p. S12-S19.
5. Birhane, N., et al., Predictors of breast self-examination among female teachers in Ethiopia using health belief model. Archives of Public Health, 2015. 73(1): p. 39.
6. Agide, F.D., et al., A systematic review of health promotion interventions to increase breast cancer screening uptake: from the last 12 years. European journal of public health, 2018.
7. Engelman, K.K., et al., Engaging diverse underserved communities to bridge the mammography divide. BMC public health, 2011. 11(1): p. 47.
8. Siegel, R., D. Naishadham, and A. Jemal, Cancer statistics, 2013. CA: a cancer journal for clinicians, 2013. 63(1): p. 11-30.
9. Coleman, M., et al., Cancer survival in Australia, Canada, Denmark, Norway, Sweden, and the UK, 1995–2007 (the International Cancer Benchmarking Partnership): an analysis of population-based cancer registry data. The Lancet, 2011. 377(9760): p. 127-138.
10. Ferlay, J., et al., Cancer incidence and mortality worldwide: sources, methods and major patterns in GLOBOCAN 2012. International journal of cancer, 2015. 136(5): p. E359-E386.
11. Bray, F., et al., Global estimates of cancer prevalence for 27 sites in the adult population in 2008. International journal of cancer, 2013. 132(5): p. 1133-1145.
12. Altobelli, E. and A. Lattanzi, Breast cancer in European Union: An update of screening programmes as of March 2014 (Review). International journal of oncology, 2014. 45(5): p. 1785-1792.
13. DeSantis, C., et al., Breast cancer statistics, 2011. CA: a cancer journal for clinicians, 2011. 61(6): p. 408-418.
14. Salem, D.S., et al., Breast imaging in the young: the role of magnetic resonance imaging in breast cancer screening, diagnosis and follow-up. Journal of thoracic disease, 2013. 5(Suppl 1): p. S9.
15. Story, H., et al., Improving outcomes from breast cancer in a low-income country: lessons from Bangladesh. International journal of breast cancer, 2011. 2012.
16. Siegel, R.L., K.D. Miller, and A. Jemal, Cancer statistics, 2016. CA: a cancer journal for clinicians, 2016. 66(1): p. 7-30.
17. Tigeneh, W., et al., Pattern of Cancer in Tikur Anbessa Specialized Hospital Oncology Center in Ethiopia from 1998 to 2010. Int J Cancer Res Mol Mech, 2015. 1: p. 1-5.
18. Alwan, N., et al., Knowledge, attitude and practice regarding breast cancer and breast self-examination among a sample of the educated population in Iraq. Eastern Mediterranean Health Journal, 2012. 18(4): p. 337.
19. Mahlet, M., Assessment Of Breast Self Examination Practice And Associated Factors Among Women Between 20 Up To 70 Years Attending On The Selected Public Health Centers In Addis Ababa, Ethiopia, 2015. 2015, Aau.
20. Lu, M., et al., A systematic review of interventions to increase breast and cervical cancer screening uptake among Asian women. BMC public health, 2012. 12(1): p. 413.
21. Corkum, M., et al., Screening for new primary cancers in cancer survivors compared to non-cancer controls: a systematic review and meta-analysis. Journal of Cancer Survivorship, 2013. 7(3): p. 455-463.
22. Sankaranarayanan, R., et al., Clinical breast examination: preliminary results from a cluster randomized controlled trial in India. Journal of the National Cancer Institute, 2011.
23. Anderson, B.O., et al., Guideline implementation for breast healthcare in low‐income and middle‐income countries. Cancer, 2008. 113(S8): p. 2221-2243.
24. Rosenstock, I.M., Historical origins of the health belief model. Health education monographs, 1974. 2(4): p. 328-335.
25. Glanz, K., B.K. Rimer, and K. Viswanath, Health behavior and health education: theory, research, and practice. 2008: John Wiley & Sons.
26. Hausmann-Muela, S., J.M. Ribera, and I. Nyamongo, Health-seeking behaviour and the health system response. Disease Control Piroirities Project working paper No14, 2003.
27. Parsa, P., et al., Barriers for breast cancer screening among Asian women: a mini literature review. Asian Pacific journal of cancer prevention, 2006. 7(4): p. 509.
28. Otto, S.J., et al., Initiation of population-based mammography screening in Dutch municipalities and effect on breast-cancer mortality: a systematic review. The Lancet, 2003. 361(9367): p. 1411-1417.
29. Baron, R.C., et al., Client-directed interventions to increase community demand for breast, cervical, and colorectal cancer screening: a systematic review. American journal of preventive medicine, 2008. 35(1): p. S34-S55.
30. Smith, R.A., V. Cokkinides, and O.W. Brawley, Cancer screening in the United States, 2009: a review of current American Cancer Society guidelines and issues in cancer screening. CA: a cancer journal for clinicians, 2009. 59(1): p. 27-41.
31. Austoker, J., et al., Interventions to promote cancer awareness and early presentation: systematic review. British journal of cancer, 2009. 101(Suppl 2): p. S31.
32. Lim, J.-w. and B. Zebrack, Different pathways in social support and quality of life between Korean American and Korean breast and gynecological cancer survivors. Quality of Life Research, 2008. 17(5): p. 679-689.
33. Jagsi, R., et al., Long-term financial burden of breast cancer: experiences of a diverse cohort of survivors identified through population-based registries. Journal of Clinical Oncology, 2014. 32(12): p. 1269-1276.
34. Darby, K., et al., Exploring the financial impact of breast cancer for African American medically underserved women: a qualitative study. Journal of health care for the poor and underserved, 2009. 20(3): p. 721-728.
35. Zimlichman, E., et al., Health care–associated infections: a meta-analysis of costs and financial impact on the US health care system. JAMA internal medicine, 2013. 173(22): p. 2039-2046.
36. Arozullah, A.M., et al., The financial burden of cancer: estimates from a study of insured women with breast cancer. J Support Oncol, 2004. 2(3): p. 271-278.
37. Zahl, P.-H., J. Mæhlen, and H.G. Welch, The natural history of invasive breast cancers detected by screening mammography. Archives of internal medicine, 2008. 168(21): p. 2311-2316.
38. Hunter, D.J., et al., A genome-wide association study identifies alleles in FGFR2 associated with risk of sporadic postmenopausal breast cancer. Nature genetics, 2007. 39(7): p. 870.
39. DeSantis, C., R. Siegel, and A. Jemal, Breast Cancer Facts & Figures 2007-2008. Am Cancer Society, 2007: p. 1-32.
40. Coughlin, S.S. and D.U. Ekwueme, Breast cancer as a global health concern. Cancer epidemiology, 2009. 33(5): p. 315-318.
41. Morris, T., H. Steven Greer, and P. White, Psychological and social adjustment to mastectomy. A two‐year follow‐up study. Cancer, 1977. 40(5): p. 2381-2387.
42. Pharoah, P.D., et al., Family history and the risk of breast cancer: a systematic review and meta‐analysis. International journal of cancer, 1997. 71(5): p. 800-809.
43. Dye, T.D., et al., Complex care systems in developing countries. Cancer, 2010. 116(3): p. 577-585.
44. Reeler, A., K. Sikora, and B. Solomon, Overcoming challenges of cancer treatment programmes in developing countries: a sustainable breast cancer initiative in Ethiopia. Clinical Oncology, 2008. 20(2): p. 191-198.
45. Remennick, L., The challenge of early breast cancer detection among immigrant and minority women in multicultural societies. The breast journal, 2006. 12(s1).
46. Jemal, A., et al., Cancer burden in Africa and opportunities for prevention. Cancer, 2012. 118(18): p. 4372-4384.
47. Jemal, A., et al., Cancer statistics, 2008. CA: a cancer journal for clinicians, 2008. 58(2): p. 71-96.
48. Moss, S.M., et al., Effect of mammographic screening from age 40 years on breast cancer mortality at 10 years' follow-up: a randomised controlled trial. The Lancet, 2006. 368(9552): p. 2053-2060.
49. Taymoori, P., Y. Molina, and D. Roshani, Effects of a randomized controlled trial to increase repeat mammography screening in Iranian women. Cancer nursing, 2015. 38(4): p. 288.
50. Fry, R.B. and S. Prentice-Dunn, Effects of a psychosocial intervention on breast self-examination attitudes and behaviors. Health Education Research, 2005. 21(2): p. 287-295.
51. Naserian, N., S. Ansari, and P. Abedi, Comparison of Training via Short Messages and Group Training on Level of Knowledge and Practice of Middle-Aged Women About Breast Cancer Screening Tests. Journal of Cancer Education, 2017: p. 1-7.
52. Rahman, S.M., et al., Perceptions related to breast cancer prevention and behavioral practices in underserved women participated in a CBPR intervention. 2014, AACR.
53. Lee, C.H., et al., Breast cancer screening with imaging: recommendations from the Society of Breast Imaging and the ACR on the use of mammography, breast MRI, breast ultrasound, and other technologies for the detection of clinically occult breast cancer. Journal of the American college of radiology, 2010. 7(1): p. 18-27.
54. Parker, J.S., et al., Supervised risk predictor of breast cancer based on intrinsic subtypes. Journal of Clinical Oncology, 2009. 27(8): p. 1160-1167.
55. Snyman, L.C., Breast cancer mammography screening for low-risk women in South Africa: letter to the editor. Southern African Journal of Gynaecological Oncology, 2010. 2(2): p. 69-70.
56. Akuoko, C.P., et al., Barriers to early presentation and diagnosis of breast cancer among African women living in sub-Saharan Africa. PloS one, 2017. 12(2): p. e0171024.
57. Adewole, I., et al., African Organisation For Research & Training In Cancer [Aortic] 2013 Edition.
58. Ramathuba, D.U., C.T. Ratshirumbi, and T.M. Mashamba, Knowledge, attitudes and practices toward breast cancer screening in a rural South African community. Curationis, 2015. 38(1): p. 1-8.
59. Lemlem, S.B., et al., Assessment of knowledge of breast cancer and screening methods among nurses in university hospitals in Addis Ababa, Ethiopia, 2011. ISRN oncology, 2013. 2013.
60. Kantelhardt, E., et al., Breast cancer survival in Ethiopia: a cohort study of 1,070 women. International journal of cancer, 2014. 135(3): p. 702-709.
61. Arshad, S., et al., Evaluating the knowledge of breast cancer screening and prevention among Arab-American women in Michigan. Journal of Cancer Education, 2011. 26(1): p. 135-138.
62. De Ver Dye, T., et al., A mixed-method assessment of beliefs and practice around breast cancer in Ethiopia: implications for public health programming and cancer control. Global public health, 2011. 6(7): p. 719-731.
63. Tefera, B., et al., Patterns of Cancer in University of Gondar Hospital: North-West Ethiopia. J Oncol Med & Pract, 2016. 1(106): p. 2.
64. Fitzmaurice, C., et al., The global burden of cancer 2013. JAMA oncology, 2015. 1(4): p. 505-527.
65. Jiagge, E., et al., Breast cancer and African ancestry: Lessons learned at the 10-year anniversary of the Ghana-Michigan research partnership and International Breast Registry. Journal of Global Oncology, 2016. 2(5): p. 302-310.
66. Lodge, M. and M. Corbex, Establishing an evidence-base for breast cancer control in developing countries. The Breast, 2011. 20: p. S65-S69.
67. Legesse, B. and T. Gedif, Knowledge on breast cancer and its prevention among women household heads in Northern Ethiopia. Open Journal of Preventive Medicine, 2014. 4(01): p. 32.
68. Motilewa, O.O., U.S. Ekanem, and C.A. Ihesie, Knowledge of breast cancer and practice of self-breast examination among female undergraduates in Uyo, Akwa Ibom State, Nigeria. 2015.
69. Organization, W.H., Training for mid-level managers. 2008.
70. Nguyen, T.T., et al., Breast cancer screening among Vietnamese Americans: a randomized controlled trial of lay health worker outreach. American journal of preventive medicine, 2009. 37(4): p. 306-313.
71. Akhtar, S., et al., First organized screening mammography programme in Saudi Arabia: preliminary analysis of pilot round/Premier programme de mammographie de depistage en Arabie saoudite: rapport preliminaire de l'operation pilote. Eastern Mediterranean Health Journal, 2010. 16(10): p. 1025.
72. Eskandari-Torbaghan, A., et al., Improving breast cancer preventive behavior among female medical staff: the use of educational intervention based on health belief model. The Malaysian journal of medical sciences: MJMS, 2014. 21(5): p. 44.
73. Tuzcu, A., Z. Bahar, and S. Gözüm, Effects of interventions based on health behavior models on breast cancer screening behaviors of migrant women in Turkey. Cancer nursing, 2016. 39(2): p. E40-E50.
74. Hajian, S., et al., Effects of education based on the health belief model on screening behavior in high risk women for breast cancer, Tehran, Iran. Asian Pac J Cancer Prev, 2011. 12(1): p. 49-54.
75. Dallo, F.J., et al., Cancer knowledge increases after a brief intervention among Arab Americans in Michigan. Journal of Cancer Education, 2011. 26(1): p. 139-146.
76. Hemming, K., et al., Sample size calculations for cluster randomised controlled trials with a fixed number of clusters. BMC medical research methodology, 2011. 11(1): p. 102.
77. Rutterford, C., A. Copas, and S. Eldridge, Methods for sample size determination in cluster randomized trials. International journal of epidemiology, 2015. 44(3): p. 1051-1067.
78. Teferi, S., et al., Knowledge about breast cancer risk-factors, breast screening method and practice of breast screening among female healthcare professionals working in governmental hospitals, Addis Ababa, Ethiopia. IOSR Journal of pharmacy and biological sciences, 2012. 2(1): p. 5-12.
79. Schulz, K.F., D.G. Altman, and D. Moher, CONSORT 2010 statement: updated guidelines for reporting parallel group randomised trials. BMC medicine, 2010. 8(1): p. 18.
80. Herman, A., et al., Intention-to-treat analysis and accounting for missing data in orthopaedic randomized clinical trials. J Bone Joint Surg Am, 2009. 91(9): p. 2137-2143.
81. Association, W.M., Declaration of Helsinki. Ethical principles for medical research involving human subjects. 2009.
