# Supplementary material for: Designing and evaluation of the effect of community-based intervention on breast self-examination among reproductive-aged women in Ethiopia: A Cluster Randomized Controlled Trial
Source: PLoS One. 2025 Aug 7;20(8):e0329839. doi: 10.1371/journal.pone.0329839 (PMC12331034; doi:10.1371/journal.pone.0329839)
Supplement: Attachment — Submitted filename: PONE-D-23-37776_Reviewed.pdf [file pone.0329839.s004.pdf]

PLOS ONE

Designing and evaluation of the effect of community-based intervention on breast self-examination among reproductive-aged women in Ethiopia: A Randomized Trial

--Manuscript Draft--

**Manuscript Number:**	PONE-D-23-37776
**Article Type:**	Clinical Trial
**Full Title:**	Designing and evaluation of the effect of community-based intervention on breast self-examination among reproductive-aged women in Ethiopia: A Randomized Trial
**Short Title:**	Designing and evaluation of community-based intervention on breast self-examination among women in Ethiopia
**Corresponding Author:**	Feleke Doyore Agide, PhD<br>WCU: Wachemo University<br>Hossana, Southern Ethiopia, Ethiopia ETHIOPIA
**Keywords:**	breast cancer; Breast Self-Examination; intervention; Randomized Trial; Exploratory Approach
**Abstract:**	Background: Though early intervention saves most lives in the world, breast cancer remained a leading cause of cancer in women in Ethiopia. Therefore, this study aimed to design and evaluate community-based interventions for breast self-examination using the health belief model.<br>Methods: This two-phase study began with a qualitative study to identify salient beliefs in the area, followed by a randomized controlled trial in six randomly selected districts of the Zone. A total of 810 participants were enrolled and evaluated at baseline, three, and six months after the intervention. A general linear model for repeated measures was used to identify the mean difference of the interventions on the variables of the study. Non-parametric tests (Cochran Q) were used for dichotomous variables to measure breast self-examination practices. Path analysis was used to see the interactions of the HBM constructs.<br>Results: The likelihood of performing breast self-examination and the comprehensive knowledge of the participants from baseline to the end of the intervention period were 33.3% to 59.9% and 11.7% to 69.1%, respectively. Perceived susceptibility, perceived severity, knowledge, and health motivations had a statistically significant mean difference between the intervention and control groups (p<0.005). This study has been registered in the Pan African Clinical Trial Registry (www.pactr.org) database, and the unique identification number for the registry is PACTR201802002902886.<br>Conclusions: The community-based intervention had a significant impact on the likelihood of performing breast self-examination. Therefore, to better understand the problem, further research is needed to assess the doze and peak of interventions where the actual behavior change is reached and provide a complete picture of conceptualizations of breast self-examination.
**Order of Authors:**	Feleke Doyore Agide, PhD
	Garmaroudi Gholamreza, PhD
	Sadeghi Roya, PhD
	Shakibazadeh Elham, PhD
	Yaseri Mehdi, PhD
	Zewdie Birhanu, PhD
**Additional Information:**	
**Question**	**Response**
**Financial Disclosure**<br><br>Enter a financial disclosure statement that describes the sources of funding for the	The author(s) received no specific funding for this work.

Powered by Editorial Manager® and ProduXion Manager® from Aries Systems Corporation

work included in this submission. Review the submission guidelines for detailed requirements. View published research articles from *PLOS ONE* for specific examples.<br><br>This statement is required for submission and **will appear in the published article** if the submission is accepted. Please make sure it is accurate.<br><br>**Funded studies**<br>Enter a statement with the following details:<br>• Initials of the authors who received each award<br>• Grant numbers awarded to each author<br>• The full name of each funder<br>• URL of each funder website<br>• Did the sponsors or funders play any role in the study design, data collection and analysis, decision to publish, or preparation of the manuscript?<br><br>Did you receive funding for this work?	
**Competing Interests**<br><br>Use the instructions below to enter a competing interest statement for this submission. On behalf of all authors, disclose any competing interests that could be perceived to bias this work—acknowledging all financial support and any other relevant financial or non-financial competing interests.<br><br>This statement is required for submission and **will appear in the published article** if the submission is accepted. Please make sure it is accurate and that any funding sources listed in your Funding Information later in the submission form are also declared in your Financial Disclosure statement.<br><br>View published research articles from *PLOS ONE* for specific examples.	The authors have declared that no competing interests exist.

Powered by Editorial Manager® and ProduXion Manager® from Aries Systems Corporation

**NO authors have competing interests**<br><br>Enter: *The authors have declared that no competing interests exist.*<br><br>**Authors with competing interests**<br><br>Enter competing interest details beginning with this statement:<br><br>*I have read the journal's policy and the authors of this manuscript have the following competing interests: [insert competing interests here]*<br><br>* typeset	
**Ethics Statement**<br><br>Enter an ethics statement for this submission. This statement is required if the study involved:<br><br>• Human participants<br>• Human specimens or tissue<br>• Vertebrate animals or cephalopods<br>• Vertebrate embryos or tissues<br>• Field research<br><br>Write "N/A" if the submission does not require an ethics statement.<br><br>General guidance is provided below. Consult the submission guidelines for detailed instructions. **Make sure that all information entered here is included in the Methods section of the manuscript.**	This trial includes human participants and stated in the methods part of the ethics.

**Format for specific study types**<br><br>**Human Subject Research (involving human participants and/or tissue)**<br>• Give the name of the institutional review board or ethics committee that approved the study<br>• Include the approval number and/or a statement indicating approval of this research<br>• Indicate the form of consent obtained (written/oral) or the reason that consent was not obtained (e.g. the data were analyzed anonymously)<br><br>**Animal Research (involving vertebrate animals, embryos or tissues)**<br>• Provide the name of the Institutional Animal Care and Use Committee (IACUC) or other relevant ethics board that reviewed the study protocol, and indicate whether they approved this research or granted a formal waiver of ethical approval<br>• Include an approval number if one was obtained<br>• If the study involved *non-human primates*, add *additional details* about animal welfare and steps taken to ameliorate suffering<br>• If anesthesia, euthanasia, or any kind of animal sacrifice is part of the study, include briefly which substances and/or methods were applied<br><br>**Field Research**<br><br>Include the following details if this study involves the collection of plant, animal, or other materials from a natural setting:<br>• Field permit number<br>• Name of the institution or relevant body that granted permission	
**Data Availability**<br><br>Authors are required to make all data underlying the findings described fully available, without restriction, and from the time of publication. PLOS allows rare exceptions to address legal and ethical concerns. See the PLOS Data Policy and FAQ for detailed information.	Yes - all data are fully available without restriction

A Data Availability Statement describing where the data can be found is required at submission. Your answers to this question constitute the Data Availability Statement and **will be published in the article**, if accepted.<br><br>**Important:** Stating 'data available on request from the author' is not sufficient. If your data are only available upon request, select 'No' for the first question and explain your exceptional situation in the text box.<br><br>Do the authors confirm that all data underlying the findings described in their manuscript are fully available without restriction?	
**Describe where the data may be found in full sentences. If you are copying our sample text, replace any instances of XXX with the appropriate details.**<br><br>• If the data are **held or will be held in a public repository**, include URLs, accession numbers or DOIs. If this information will only be available after acceptance, indicate this by ticking the box below. For example: *All XXX files are available from the XXX database (accession number(s) XXX, XXX.).*<br>• If the data are all contained **within the manuscript and/or Supporting Information files**, enter the following: *All relevant data are within the manuscript and its Supporting Information files.*<br>• If neither of these applies but you are able to provide **details of access elsewhere**, with or without limitations, please do so. For example:<br><br>*Data cannot be shared publicly because of [XXX]. Data are available from the XXX Institutional Data Access / Ethics Committee (contact via XXX) for researchers who meet the criteria for access to confidential data.*<br><br>*The data underlying the results presented in the study are available from (include the name of the third party*	All relevant data are within the manuscript and its Supporting Information files.

and contact information or URL).<br>• This text is appropriate if the data are owned by a third party and authors do not have permission to share the data.<br><br>* typeset	
Additional data availability information:	

Manuscript

Click here to access/download;Manuscript;Main Manuscript final PLOSE One.docx

Designing and evaluation of the effect of community-based intervention on breast self-examination among reproductive-aged women in Ethiopia: A Randomized Trial

Feleke Doyore Agide[1, 2*], Gholamreza Garmaroudi[3], Roya Sadeghi[3], Elham Shakibazadeh[3], Mehdi Yaseri[4], Zewdie Birhanu[5]

[1]Department of Health Education and Promotion, School of Public Health, International Campus, Tehran University of Medical Sciences, Tehran, Iran.

[2]Department of Public Health, College of Medicine and Health Sciences, Wachemo University, Hossana, Ethiopia.

[3]Department of Health Education and Promotion, School of Public Health, Tehran University of Medical Sciences, Tehran, Iran.

[4]Department of Epidemiology and Biostatistics, School of Public Health, Tehran University of Medical Sciences, Tehran, Iran.

[5]Department of Health, Behavior and Society, Institute of Health Sciences, Jimma University, Jimma, Ethiopia.

Registered in the Pan African Clinical Trial Registry (www.pactr.org) database, and the unique identification number for the registry is **PACTR201802002902886.**

***Corresponding Author:**

E-mail: feledoag@yahoo.com (FDA)

Authors' contribution:

FDA, GG, RS, ES, MY and ZB were involved in the design, conduct and analysis of the study. FDA prepared the manuscript for publication and all authors critically reviewed the manuscript.

Abstract

Background: Though early intervention saves most lives in the world, breast cancer remained a leading cause of cancer in women in Ethiopia. Therefore, this study aimed to design and evaluate community-based interventions for breast self-examination using the health belief model.

Methods: This two-phase study began with a qualitative study to identify salient beliefs in the area, followed by a randomized controlled trial in six randomly selected districts of the Zone. A total of [illegible] participants were enrolled and evaluated at baseline, three, and six months after the intervention. A general linear model for repeated measures was used to identify the mean difference of the interventions on the variables of the study. Non-parametric tests (Cochran Q) were used for dichotomous variables to measure breast self-examination practices. Path analysis was used to see the interactions of the HBM constructs.

Results: The likelihood of performing breast self-examination and the comprehensive knowledge of the participants from baseline to the end of the intervention period were 33.3% to 59.9% and 11.7% to 69.1%, respectively. Perceived susceptibility, perceived severity, knowledge, and health motivations had a statistically significant mean difference between the intervention and control groups ($p<0.005$). This study has been registered in the Pan African Clinical Trial Registry (www.pactr.org) database, and the unique identification number for the registry is PACTR201802002902886.

Conclusions: The community-based intervention had a significant impact on the likelihood of performing breast self-examination. Therefore, to better understand the problem, further research is needed to assess the doze and peak of interventions where the actual behavior change is reached and provide a complete picture of conceptualizations of breast self-examination.

Keywords: **Breast Cancer, Breast Self-Examination, Intervention, Randomized Trial**

Introduction

Breast cancer (BC) is a non-communicable disease where ductal carcinoma starts in the tubes (ducts) that carry milk from the breast to the nipple through the development of cells that begin to grow out of control [1-3]. According to the 2020 global estimation, breast cancer affects about 2.3 million women annually and is the leading cause of cancer-related deaths among women in the developing and developed worlds [1, 2]. Though its prevalence is higher in the developed world, the rate in developing countries is increasing intolerably, including Ethiopia [4]. In various research reports, risk factors for breast cancer related to lifestyle, such as eating unhealthily, being obese, and using toxic substances, are linked to higher morbidity and mortality rates [4, 5]. Furthermore, the incidence is increasing in developing countries as a result of increased longevity, urbanization, and the adoption of Western lifestyles [6, 7].

The timing of detection determines the effectiveness of breast cancer treatment [8, 9]. Ethiopian women typically present for care at a late stage in the disease, where treatment is most ineffective, and while system-related barriers to care account for a portion of that delay in access, a lack of awareness of breast cancer symptoms accounts for a stalled initiation of action [8]. For this reason, early breast screening through breast self-examination is the preferred method for early detection of breast cancer. Previous literature documented breast self-examination, and early detection had shown improvement in the treatment outcome of breast cancer [7, 8].

In Ethiopia, despite various breast cancer prevention mechanisms suggested by health professionals, early recognition of the symptoms and self-referral for treatment are still in question, and their chances of survival are nil due to a late report [4, 8-10]. Breast self-examination has a significant impact on early diagnosis and prompt care [10]. Several observational studies have been conducted on breast self-examination among women, but none

of these were interventional studies among women of reproductive age in Ethiopia [11–13]. Thus, health promotion interventions have immense potential for awareness creation, early detection, and improved survival [14–16].

Therefore, the aim of this study was to design and evaluate community-based interventions on breast self-examination that used CONSORT flow diagram of a randomized trial of two groups (Figure 1) in the theoretical framework of the Health Belief Model (HBM). HBM is a socio-psychological model that attempts to explain and predict health behaviors in terms of certain belief patterns by focusing on the attitudes and beliefs of individuals. It was developed by social psychologists to explain the lack of public participation in health screening and prevention programs. Since then, it has been adapted to a variety of long- and short-term health behaviors, including breast screening behaviors [17]. The HBM addresses the individual‘s perceptions of the threat posed by a health problem (susceptibility, severity), the benefits of avoiding the threat, and factors influencing the decision to act (barriers, cues to action, and self-efficacy); it also states specific health beliefs related to the health problem and recommended health actions that influence the likelihood of taking recommended health actions (breast self-examination) [14, 17-20] (Figure 2).

Materials and Methods

Study setting: This study was conducted on women of reproductive age in the Hadiya zone of Southern Ethiopia. There are 10 rural districts, two city administrations, and 332 kebeles and Kifleketemas within the zone. Hossana, its capital city, is 230 kilometers from Addis Abeba. The zone's population is expected to be 1,850,104 people. Females of childbearing age (15-49) are estimated to be 193,967. The overall number of health institutions by kind in the zone correlates to kebele numbers and more. Health extension workers and community health agents play an important role in the prevention of communicable and non-communicable diseases. The research was carried out between April 2018 and April 2019.

Study design and populations: This two-phase study began with a qualitative study to identify salient beliefs in the area, followed by a randomized controlled trial in six randomly selected districts in Ethiopia. A total of 810 participants were enrolled and evaluated at baseline, three, and six months after the educational intervention. Then, a randomized controlled trial followed by a cross-sectional study lasting six months was used to evaluate the effectiveness of the community-based educational intervention on breast self-examination among women of reproductive age in Ethiopia. The study included women in the reproductive age group (15-49) who were physically and mentally competent of providing informed consent, could follow the intervention provided without assistance, and were ready to give data to the researcher. Exclusion criteria included individuals who could not stay until the intervention was finished, people who could move during the intervention, and those who could not attend more than two training sessions. For the qualitative part, at the beginning, an exploratory qualitative study with

women and health workers was conducted and published elsewhere, utilizing focus group discussions (FGD) and in-depth interviews, respectively.

Recruitment, sampling and sample size determination: Hadiya Zone has twelve districts (ten rural districts and two city administrations at the time of data collection). For sampling, we selected six districts randomly. Then, we selected 30 kebeles from the selected districts, and we were evenly distributed in intervention and control groups (i.e., 30 kebeles were divided into 15 control and 15 intervention groups). A systematic sampling was used to select each participant from each kebele by adding every K value in the first households. To prevent information contamination, random assignment was used to make the control and interventional sites far from each other. We divided 810 participants proportionally for each selected kebele. Hence, the total number of study kebeles was based on the WHO sampling recommendation (WHO) [21]. For participant enrollment, women who were potentially eligible for the study were listed, and each individual was invited to participate in the study through an oral invitation made by the principal investigator and health extension workers of each selected kebeles. An appointment was made if the person agreed to participate. Following oral informed consent, women were included for eligibility as subjects of the study, baseline data was obtained, and participants were allocated either an intervention or control group. Oral informed consent was obtained from the women who agreed to participate in the study. Participants in the randomized controlled trial were assigned 1:1 to the intervention and control arms under a restricted randomized design after informed consent and the collection of baseline data. The different kebeles were alphabetically coded (A, B, C, D, and so on), and participants attached to each kebele were given numerical codes (for example, participants in kebele 1 were numbered 1001, 1002, 1003, and so on). Interventionists, data collectors, and statisticians were not the same people. The baseline data

collection recruitment began on April 1, 2018. Then, on May 7, 2018, the first education intervention was delivered and continued for three months, every 15 days. The follow-up data was collected from both groups after three months and at the end of six months. To inform you of a deviation from the initial protocol, we confirm that the initial protocol was prepared for all breast screening behaviors (breast self-examination, breast clinical exam, and mammography use as a sequential exploratory study including qualitative and quantitative as a PhD thesis, and the qualitative and baseline were published elsewhere). The data collection period was a bit longer than stated in the initial protocol.

Concerning sample size determination, since this study is a cluster randomized trial, the sample size was calculated using the double population proportion formula by considering 77.6% of the participants who have knowledge about breast screening methods (P1 = 77.6%) [22]; P2 is the prevalence of screening rate in the intervention districts (87.6%) (assumed to be increased by 10%); K is the coefficient of variation of true proportions of the outcome variable between the districts within each group; margin of error 5%, a 5% level of significance (two-sided) i.e. 95% confidence interval of certainty. Since there is no study to estimate k, it is taken as 0.25. Then, the sample size was 368. Finally, the sample size was further increased by 10% to account for contingencies such as non-response or recording error, i.e., 368 X 10/100 + 368 = 404.8 ≈ 405. Therefore, the final sample size was 810 due to the design effect.

Measurement and variables: The likelihood of performing breast self-examination is the intended outcome for this study (perceived benefits minus perceived barriers). The exposure variables were socio-demographic factors, knowledge of breast cancer and its screening methods, and past behaviors related to screening. Socio-demographic characteristics include age, marital status, religion, place of residence, educational and occupation status, and with whom the

respondents are currently living. There are knowledge questions with a response format of ‘yes’ or ‘no’. Respondents were asked not to guess but to mark the ‘I don‘t know’ answer possibility if they did not know the correct answer. Knowledgeable are those respondents who answered 50% or more of all the knowledge questions about breast cancer and its screening methods. Not knowledgeable were those respondents who could answer below 50% of all the knowledge questions about breast cancer and its screening methods. Perceived susceptibility is the respondent‘s self-perception of vulnerability to breast cancer, measured by the summed score of related belief items on a 5-point Likert scale. Perceived severity of breast cancer is the respondent‘s held belief concerning the effects of breast cancer's seriousness, measured by the summed score of related belief items on a 5-point Likert scale. Perceived benefits of breast self-examination are the respondent‘s beliefs about the effectiveness of the method as a strategy for breast cancer prevention, measured by the summed score of related belief items on a 5-point Likert scale. Perceived barriers to performing breast self-examination are the respondent‘s beliefs about the ease of performing the given preventive action. Self-efficacy is the respondent‘s self-confidence to perform breast self-examination by herself in any condition and elsewhere to prevent breast cancer, as measured by the summed score of related belief items on a 5-point Likert scale. Negatively worded items were reversed before calculating a summed score for each concept. Cues to actions are conditions that might facilitate them to perform breast self-examination in the respondents’ surroundings with a response format of ‘yes or no’. Past behavior (practice) is the experience of the reproductive-aged women to undergo breast self-examination at least once in the recommended period to prevent breast cancer, measured with nominal measurements.

Procedures (Intervention Description): An educational intervention was prepared based on health belief model constructs, which are interlinked with breast self-examination. The intervention emerged from qualitative parts that were treated as distal factors in the model and used as a very important base for intervention design. Educational intervention was provided on breast self-examination through training and teaching using different methods and materials, like posters. Concerning the process, the enumerated lists of the participants were secured from the registry book of health workers after getting consent. Immediately after baseline data collection, the participants were categorized into intervention and control groups. All the required information for both groups was taken, registered on a temporarily prepared attendance sheet, and followed accordingly. Participants in the intervention arm received community-based educational intervention every 15 days for 3 months and registered their names and phone numbers (even family phone numbers) for tracking and reminder purposes. All of the participants were promised confidentiality throughout the process. The participants were given an appointment at a health center or health post near them where the usual community forum was being conducted. The interventionists, together with health extension workers, delivered community-based interventions. The control group received the usual services from health extension workers. These participants only received a welcome message at the beginning to validate their entry into the study and a message at the end of the follow-up to thank them for their participation. However, at the end of the six months, the same education was provided for the controlled groups.

Data collection, quality management, processing, and analysis: Data were collected using designed and adapted, structured interviewer-administered questionnaires. Questionnaires were translated into the local language and then back translated to English by another person to

maintain consistency. Two days of training were given for data collectors and supervisors. Supervisors and the principal investigator performed immediate supervision on a daily basis. The data were analyzed by SPSS V. 24.0. Prior to analysis, the data were checked for normality and homogeneity, then analyzed and interpreted by the research team and a biostatistician. Intervention outcomes were analyzed following the reporting standards of the consolidated standard of reporting trials (CONSORT standards) (Figure 1). For intervention and control arms comparison, a rate of breast self-examination at baseline, after three months, and at the end of six months was compared by using Chi-square and ANOVA. A general linear regression model for repeated measures was used to assess the effectiveness of the intervention and predict independent predictors of self-examination. Non-parametric tests (Cochran Q) were used for dichotomous variables to measure the effect size of the intervention on breast self-examination. Path analysis was used to determine the direct and indirect effects of variables and to estimate the values of coefficients in the underpinning linear model at the end of six months.

Ethics: Ethical approval was obtained from the research ethical review and approval board (RERB) of Tehran University of Medical Sciences, International Campus (TUMS-IC: (IR.TUMS.SPH.REC.1396.4088)) and the Research and Ethical Review Approval Committee of Southern Ethiopia Regional Health Bureau (RERC: 6-19/5524). This study also strictly followed the ethical principles of the Helsinki Declaration of Medical Research (Association 2009). The letters written from TUMS-IC and the Southern Ethiopia Regional Health Bureau were given to the Hadiya Zone Health Department for legal permission. Informed verbal consent was obtained from each participant after thoroughly explaining the objectives and benefits of the study. The study subjects had the right to withdraw at any time from the study. The participants were also informed that their responses would be kept confidential and that their names would not be

mentioned. At the end of the data collection for the evaluation part, the same education was provided for the controlled groups. This study has been registered in the Pan African Clinical Trial Registry (www.pactr.org) database, and the unique identification number for the registry is PACTR201802002902886.

Results

Baseline and socio-demographic characteristics of the participants:

Eight hundred and ten reproductive-aged women participated in the study, giving a response rate of 100% at baseline. The baseline data were collected from both groups before the commencement of the intervention and control groups. Then after three and six months of intervention, the same data were collected again to see the effect of the intervention. Table 1 shows the socio-demographic characteristics of the participants at baseline. Accordingly, at the baseline survey, the mean age of the participants was 33.2 (SD ±7.7) and 33.5 (SD ± 8.1) years in the intervention and control arms, respectively. Accordingly, a total of 810, 792 and 778 reproductive-aged women participated at baseline, three, and six months of the study, respectively. The intervention and control districts had no statistically significant differences in terms of age, marital status, place of residence, religion, or monthly income ($P>0.05$) (Table 1).

Table 1: Socio-demographic characteristics of the participants at baseline

Variables	Category	Intervention and Control Categories (Measurement Time)				
		Baseline				
		Intervention group (n=405)		Control group (n=405)		P –value
		No.	%	No.	%	
Age	15-24	80	19.8	92	22.7	**0.343**
	25-34	166	41.0	141	34.8	
	35-44	136	33.6	129	31.9	
	45-49	23	5.7	43	10.6	
Current Residence	Rural	205	50.6	320	79.0	0.427
	Urban	200	49.4	85	21.0	
Religion	Protestant	289	71.4	308	76.0	0.687
	Orthodox	70	17.3	71	17.5	
	Muslim	31	7.7	14	3.5	
	Catholic	15	3.7	12	3.0	
Marital status	Single	32	7.9	45	11.1	0.162
	Married	350	86.4	344	84.9	
	Divorced	23	5.7	16	4.0	
Educational Status	Can't read and write	265	65.4	188	46.4	0.000*
	Can read and write	95	23.5	102	25.2	
	Primary school	11	2.7	30	7.4	
	High school	14	3.5	45	11.1	
	College and above	20	4.9	40	9.9	
Occupational status	House wife	282	69.6	285	70.4	0.045*
	Employee	36	8.9	28	6.9	
	Merchant	28	6.9	39	9.6	
	Private business	33	8.1	32	7.9	
	Students	26	6.4	21	5.2	
Categorized Income	<= 500	282	69.6	268	66.2	0.353
	501-1000	75	18.5	87	21.5	
	1001-1500	9	2.2	22	5.4	
	> 1500	39	9.6	28	6.9	

Knowledge about breast cancer and breast self-examination

Figure 3 shows the knowledge of the participants about breast cancer and breast self-examination in the intervention and control groups across the study period. Accordingly, the study revealed that the mean of the comprehensive knowledge of the participants shifted from baseline 4.0±1.82 to 13.0±1.62 and 13.0±1.79 after three months and six months of the intervention, respectively. However, in the control group, the mean (4.0±2.11) showed a slight increase after three months and at the end of the intervention, whereas there was no significant difference between the two interventions (6±1.75 and 6±1.78) (Figure 3).

Past behaviors (practice) related to breast self-examination and its screening procedure

Table 2 presents the past behavior of the participants about breast self-examination and its screening procedure. Accordingly, almost all (95.6%) (94.6% of the participants in the intervention group and 97.0% of the control group) heard of breast cancer at baseline. Regarding screening methods, 36.9% (299/810) of participants had heard of breast self-examination as a method at baseline. However, after intervention, in the intervention group, all the participants heard of breast self-examination as a method (Table 2).

[illegible]ble 2: Descriptive frequencies and chi-square tests of past behaviors related to breast cancer and its screening among reproductive aged women in Ethiopia.

**Variables**		**Categories**	**Measurement time (Intervention and control categories)**												***P***
			**Baseline**				**After three months**				**After six months**				
			**Intervention group**		**control group**		**Intervention group**		**control group**		**Intervention group**		**control group**		
			**No**	**%**	**No**	**%**	**No**	**%**	**No.**	**%**	**No.**	**%**	**No.**	**%**	
Ever heard breast cancer before?		Yes	383	94.6	391	97.0	393	100.0	394	98.7	382	100.0	391	98.7	0.006
		No	22	5.4	14	3.0	0	0.0	5	1.3	0	0.0	5	1.3	
Ever heard screening methods?		Yes	161	39.8	138	34.1	393	100.0	142	35.3	382	100.0	140	35.4	0.000
		No	244	60.2	267	65.9	0	0.0	258	64.7	0	0.0	256	64.6	
Source of information	Health Worker	Yes	157	97.5	119	86.2	393	100.0	124	87.7	382	100.0	122	87.1	0.000
	Media	Yes	99	61.5	84	60.9	177	45.0	72	50.7	173	45.3	71	50.7	0.000
	Relative	Yes	99	61.5	84	60.9	139	35.4	91	64.1	135	35.3	89	63.6	0.000
	Friends	Yes	81	50.3	59	42.8	167	42.5	68	47.9	164	42.9	67	47.9	0.423
Ever screened your breast before?		Yes	47	29.2	23	17.2	107	27.2	32	22.5	99	25.9	22	15.7	0.000
Time of screening		2 Months Before	5	10.6	2	8.7	7	6.5	2	6.3	7	7.1	2	8.7	0.000
		6 Months Before	29	61.7	9	39.1	64	59.8	9	28.1	60	60.6	5	21.7	
		A year ago	13	27.7	9	39.1	36	33.6	19	59.4	32	32.3	14	60.9	
		2 years ago	0	0.0	3	13.0	0	0.0	2	6.3	0	0.0	2	8.7	
Method of screening used?		BSE	32	68.1	15	65.2	95	88.8	25	78.1	90	90.9	18	78.3	
		BCE	12	25.5	5	21.7	9	8.4	5	15.6	9	9.1	5	21.7	0.000
		Mammography	3	6.4	3	13.0	3	2.8	2	6.3	0	0.0	0	0.0	
Frequency of breast screening		Sometimes	27	57.4	13	56.5	65	60.7	19	59.4	57	57.6	12	52.2	0.000
		Usually	1	2.1	2	8.7	5	4.7	2	6.3	5	5.1	2	8.7	
		Consistently	4	8.5	3	13.0	16	15.0	7	21.9	16	16.2	6	26.1	
		Others	15	31.9	5	21.7	21	19.6	4	12.5	21	21.2	3	13.0	

Perception towards breast cancer and breast self-examination

Table 3 summarizes the perception scores of the participants about breast cancer and breast self-examination. Accordingly, the likelihood of performing breast self-examination and the comprehensive knowledge of the participants were 31.3% and 9.5% at baseline, respectively. However, the likelihood of performing breast self-examination and the comprehensive knowledge of the participants were 58.8% and 66.1% at the end of the intervention, respectively. Likewise, perception of threat appraisals (i.e., perceived susceptibility to and perceived severity of breast cancer) had respective average scores of (mean ± standard deviation) in the intervention (17.6±4.5 and 39.7±8.9) and control group (17.2±4.8and 38.7±9.5) at baseline. However, there was a significant mean difference after intervention and at the end of six months ($P<0.05$). As far as the likelihood of performing breast self-examination is concerned, the perceived benefits and barriers of breast self-examination had respective average scores of (mean ± standard deviation) and in the intervention (53±10.0 and 71±10.4) and control groups (53±9.8 and 68±10.9) at baseline. However, there was a statistically significant mean difference after three months of intervention and at the end of six months in the intervention group (60±4.29 and 60±4.35, respectively) as compared to the control group ($P<0.05$) (Table 3).

Table 3: Mean scores of perception, health motivations and knowledge of the participants about breast cancer and its screening at baseline, after three months and after six months of intervention

Variables	Score ranges	Intervention and Control Categories (Measurement time)								
		Baseline			After three months			After six months		
		Intervention	Control	*P-value*	Intervention	Control	*P-value*	Intervention	Control	*P-value*
		Mean±SD	Mean±SD		Mean±SD	Mean±SD		Mean±SD	Mean±SD	
Perceived Susceptibility	5-25	17.6±4.5	17.2±4.8	P>0.05	20.7±2.2	17.1±4.8	0.000	20.8±2.1	17.0±4.8	0.000
Perceived Severity	11-55	39.7±8.9	38.7±9.5	P>0.05	50.0±2.6	43.2±7.7	0.000	50.2±2.5	43.5±7.5	0.000
Perceived Benefits	14-70	53.0±10.0	53.0±9.8	P>0.05	60.0±4.3	54.0±9.6	0.000	60.0±4.4	54.0±9.5	0.000
Perceived Barrier	19-95	71.0±10.4	68.0±10.9	P<0.05	81.0±4.4	67.0±11.6	0.000	81.0±4.3	67.0±11.6	0.000
Self-efficacy	13-65	47.0±9.5	46.5±10.5	P<0.05	55.6±3.8	46.8±10.3	0.000	55.6±3.6	46.8±10.3	0.000
Cues to action	0-7	3.5±2.0	4.0±1.9	P>0.05	4.6±1.9	4.02±1.9	0.000	4.6±1.9	4.0±1.9	0.000
Health Motivation	12-60	48.0±5.5	47.0±6.1	P>0.05	51.0±3.8	41.0±8.1	0.000	53.0±3.1	47.0±5.5	0.135
Knowledge	0-17	4.0±1.8	4.0±2.1	P>0.05	13.0±1.6	6.0±1.8	0.000	13±1.8	6±1.8	0.000
Comprehensive knowledge	0-17	9.5	9.5	p>0.05	66.3	11.3	0.000	66.1	11.3	0.000
Likelihood of BSE	Weighted mean	31.3	30.2	p>0.05	59.1	32.6	0.000	58.8	32.5	0.000

The independent predictors of likelihood of performing breast self-examination (Regression analysis)

A general linear model of repeated measures was used to assess the effect of interventions on the variables of the study. Table 4 presents the General Linear Regression Model analysis of repeated measures to compare the mean difference (two-way repeated measures of ANOVA). Accordingly, health belief model constructs, knowledge, and health motivations had a statistically significant mean difference between intervention and control groups ($p<0.005$). Likewise, the mean score of perceived barriers was statistically significantly reduced in the intervention group after three and six months (mean difference = -3.697 between time 1 and time 2 and -3.495 between time 2 and time 3). However, the mean difference in cues to action below one indicated the least variance explained by the intervention in the given situation (Table 4).

Table 4: General regression model analysis for repeated measure to see the effect (Mean Difference) of the intervention in outcome

Variables	Times		Mean Difference (Intervention Vs control)	Standard Error	P-value	95% Confidence Interval for mean difference	
						Lower Bound	Upper Bound
Susceptibility score	Time 1	Time 2	1.415	0.209	0.000	0.915	1.916
		Time 3	1.383	0.219	0.000	0.856	1.909
Severity score	Time 1	Time 2	7.446	0.377	0.000	6.541	8.351
		Time 3	7.607	0.380	0.000	6.696	8.519
Benefits score	Time 1	Time 2	3.600	0.436	0.000	2.553	4.647
		Time 3	3.505	0.442	0.000	2.445	4.565
Barriers score	Time 1	Time 2	-3.697	0.494	0.000	-4.883	-2.511
		Time 3	-3.495	0.510	0.000	-4.717	-2.272
Self-efficacy score	Time 1	Time 2	4.475	0.457	0.000	3.379	5.571
		Time 3	4.476	0.455	0.000	3.383	5.568
Health motivation score	Time 1	Time 2	1.227	0.318	0.000	0.464	1.990
		Time 3	2.663	0.260	0.000	2.039	3.287
Cues to action score	Time 1	Time 2	0.544	0.098	0.000	0.308	0.780
		Time 3	0.560	0.094	0.000	0.334	0.785
Knowledge score	Time 1	Time 2	5.545	0.099	0.000	5.307	5.783
		Time 3	5.529	0.095	0.000	5.302	5.756

Based on estimated marginal means; b. Adjustment for multiple comparisons: Bonferroni.

Effects intervention on each constructs (perceptions of variance explained)

Table 5 presents the effect of community-based interventions on each dependent variable (variance explained by the interventions). Accordingly, the community-based intervention explained 81.0% of the variance in knowledge with a statistically significant impact on the intervention group (p=0.000). The program also had a statistically significant effect on health motivation, explaining 43.1% of the variance (p=0.000). Regarding threat appraisal, the intervention explained the variance of 21.7% on perceived susceptibility to and 24.5% severity of the breast cancer having a statistically significant impact on the intervention group (p=0.000). However, the program explained the least variance (1.1%) in cues to action with a statistically significant impact on the intervention group (p=0.004) (Table 5).

Table 5: General regression model analysis for repeated measures after adjustment for multiple comparisons (ANOVA) to see the effect of intervention on the program (dependent variable)

Variables	Source	Df	Mean Square	F	Sig.	Partial Eta Squared
**Knowledge score**	Intercept	1	136400.193	35937.169	0.000	0.979
	Intervention	1	12568.878	3311.505	0.000	0.810
**Susceptibility**	Intercept	1	783664.873	47879.105	0.000	0.984
	Intervention	1	3526.629	215.464	0.000	0.217
**Severity**	Intercept	1	4506599.604	86642.524	0.000	0.991
	Intervention	1	13127.782	252.391	0.000	0.245
**Benefit**	Intercept	1	7172784.850	109005.975	0.000	0.993
	Intervention	1	13064.192	198.539	0.000	0.204
**Barrier**	Intercept	1	12199491.540	147041.048	0.000	0.995
	Intervention	1	58938.209	710.385	0.000	0.478
**Self-efficacy**	Intercept	1	5709364.740	72790.201	0.000	0.989
	Intervention	1	20452.199	260.750	0.000	0.252
**Cues to action**	Intercept	1	39533.395	10649.094	0.000	0.932
	Intervention	1	31.082	8.372	0.004	0.011
**Health motivation**	Intercept	1	5339091.815	182773.287	0.000	0.996
	Intervention	1	17174.261	587.927	0.000	0.431

*Bonferroni Tests of Between-Subjects Effects of pairwise comparisons at crude level

Effect of interventions on likelihood of performing breast self-examination

The actual breast screening behavior was assessed as a past behavior. Table 6 presents the effect size measured for dichotomous variables on screening behavior by the intervention using nonparametric tests (Cochran Q). Accordingly, the effects of intervention were demonstrated by hearing about breast cancer and performing breast self-examination at different time points or under different conditions, which had a statistically significant effect on the study population (p=0.000). However, in the case of friends as source information, it indicated that the percentages or proportions at the different time points or under the different conditions are the same in the study population. A[illegible] far as performing breast self-examination was concerned, the effect of the intervention had a statistically significant effect on breast self-examination (p=0.001) and produced higher percentages than all other behaviors. Concerning the source of information, the exposure of participants to media and health worker information significantly increased after intervention and was maintained at the maintenance stage (six months) (p=0.000). However, the intervention had no significant effect on producing exposures to friends' messages (P=0.413). The frequency of breast screening had significantly increased after intervention (at least sometimes) in the last six months (p=0.000) (Table 6). However, breast screening behavior immediately after intervention had no significant effect on screening.

Table 6: Effect size measured for dichotomous variables on screening behavior by the intervention using non-parametric tests (Cochran Q)

Variables	Categories	Measurement of time (Breast screening behavior)							
		Baseline		At 3 months		At 6 months		Effect size	
		Intervention n(%)	Control n(%)	Intervention n(%)	Control n (%)	Intervention n(%)	Control n(%)	At 3 months n(%)	At 6 months n(%)
Ever heard BC?	Yes	383 (94.6)	391(97.0)	393 (100.0)	394(98.7)	382(100.0)	391(98.7)	22.5(p=0.000)	22.5(p=0.000)
Heard screening methods?	Yes	161 (39.8)	138(34.1)	393(100.0)	142(35.3)	382(100.0)	140(35.4)	140.1(p=0.000)	132.2(p=0.000)
Source of information	Health worker	157 (97.5)	119(86.2)	393(100.0)	124(87.7)	382(100.0)	122(87.1)	95.0 (p=0.025)	95.0 (p=0.025)
	Media	99 (61.5)	84 (60.9)	177 (45.0)	72(50.7)	173 (45.3)	71 (50.7)	62.2(p=0.000)	61.7(p=0.000)
	Relative	99 (61.5)	84(60.9)	139 (35.4)	91(64.1)	135 (35.3)	89 (63.6)	75.1 (p=0.000)	74.1(p=0.000)
	Friends	81 (50.3)	59 (42.8)	167 (42.5)	68 (47.9)	164 (42.9)	67(47.9)	0.7 (p=0.413)	0.7(p=0.413)
BC screened	Yes	47 (29.2)	23 (17.2)	107 (27.2)	32 (22.5)	99 (25.9)	22 (15.7)	70.0 (p=0.000)	70.0 (p=0.000)
Method of screening used?	Mammography	3(6.4)	3(13.0)	3(2.8)	2 (6.3)	6 (10.6)	5 (21.0)	3.0 (P=0.083)	1.0 (P=0.317)
	BCE	12(25.5)	5 (21.7)	9 (8.4)	5(15.6)	9 (9.1)	5 (21.7)	3.0 (P=0.083)	3.0 (P=0.083)
	BSE	32 (68.1)	15 (65.2)	95 (88.8)	25 (78.1)	90 (90.9)	18 (78.3)	73.0 (p=0.000)	61.0 (p=0.000)
Time of screening	2 months Before	5 (10.6)	2(8.7)	7 (6.5)	2(6.3)	7(7.1)	2 (8.7)	2.0 (P=0.157)	2.0 (P=0.157)
	6 months Before	29 (61.7)	9 (39.1)	64 (59.8)	9(28.1)	60 (60.6)	5 (21.7)	35.0 (p=0.000)	27.0 (p=0.000)
	A year ago	13 (27.7)	9 (39.1)	36 (33.6)	19(59.4)	32 (32.3)	14 (60.9)	33.0 (p=0.000)	24.0 (p=0.000)
	2 years ago	0 (0.0)	3(13.0)	0 (0.0)	2(6.3)	0 (0.0)	2(8.7)	1.0 (p=0.317)	1.0 (p=0.317)
Frequency of breast screening	Sometimes	27 (57.4)	13 (56.5)	65 (60.7)	19 (59.4)	57 (57.6)	12 (52.2)	44.0 (p=0.000)	29.0 (p=0.000)
	Usually	1(2.1)	2(8.7)	5(4.7)	2 (6.3)	5 (5.1)	2 (8.7)	4.0 (p=0.046)	4.0 (p=0.046)
	Consistently	4 (8.5)	3(13.0)	16 (15.0)	7 (21.9)	16 (16.2)	6 (26.1)	16.0 (p=0.000)	15.0 (p=0.000)
	Others(once, ill)	15 (31.9)	5(21.7)	21 (19.6)	4 (12.5)	21(21.2)	3 (13.0)	5.0 (p=0.025)	4.0 (p=0.046)

The interactions/effects of HBM Constructs on likelihood of screening behavior

Path analysis was done to determine the direct and indirect effects of variables (HBM) and to estimate the values of coefficients in the underpinning linear model. Path analysis is simply standardized partial regression coefficient partitioning the correlation coefficients into measures of direct and indirect effects of a set of independent variables on the dependent variable. Identification of the model refers to how many things we have to estimate (such as the path coefficients and correlations) in relation to how much information we can derive from the data or information in terms of the observed variances of the variables and the covariance among them. In this regression model, the amount of information is exactly equal to the number of paths we have to estimate; it is just identified. Table 7 presents a path analysis to see the interaction effect on HBM constructs. Accordingly, the path coefficients for the full model (with all the arrows) were derived from a series of layered multiple regression analyses. For each multiple regression, the criterion is the variable in the box (all boxes after the leftmost layer), and the predictors are all the variables that have arrows leading to that box. Since our intention was to see the relationship between the variables, considering the coefficients or corresponding number of coefficients deprived showed the magnitude in which the arrows are explaining or imposing the effect on the likelihood of taking action. The greater estimation was demonstrated in perceived benefits and barriers (Table 7).

Table 7: Direct and indirect effects obtained by Path Analysis Model to see the interactions/effect on likelihood of taking action

Path predictor Variables/Path Through	Causal Effect		
	Direct	Indirect	Total
Perceived susceptibility with likelihood of breast screening	0	0.02*0.54	0.01
Perceived severity with likelihood of breast screening	0	0.08*0.54	0.04
Perceived benefits with likelihood of breast screening	0.72*0.54	0	0.39
Perceived barriers with likelihood of breast screening	0.54*0.54	0	0.29
Self-efficacy with likelihood of breast screening	0.06*0.54	0	0.03
Perceived threat with likelihood of breast screening	0.15*0.54	0	0.08
Cues to action with likelihood of breast screening	0	0.04*0.54	0.02
Health motivation with likelihood of breast screening	0	0.05*0.54	0.03

Discussion

This sequential exploratory study estimated the likelihood of performing breast self-examination under the basic constructs of HBM [23]. Qualitative and cross-sectional studies at baseline found that there was a strong interplay between perception and breast screening (published elsewhere). Accordingly, the study found that the likelihood of performing breast self-examination and the comprehensive knowledge of the participants were 33.3% and 11.7%, respectively. After intervention (RCT findings), the likelihood of performing breast self-examination and the comprehensive knowledge of the participants were 59.9% and 69.1% at the end of six months, respectively. In countries where the majority of the women are not educated (like Ethiopia), repeated education has a significant impact on reproductive-aged women, at least in producing the intention to have breast self-examination. The study also confirmed that community-based intervention had a significant effect on increasing the likelihood of performing breast self-examination.

In this study, the intervention increased the perception as well as the actual screening practice of the reproductive-aged women. However, the effect of intervention was either maintained or a bit reduced at the maintenance point (after six months), indicating sustained education is needed to have a healthy implication on the given behavior. Similarly, a South African study shows uptake of screening among women after offering education increased [24, 25]. This is also supported by the concept of health promotion, which believes education is not a one-time affair but rather a continuous process [17, 23].

Previous studies have documented that interventions have a considerable effect on increasing the awareness of a large audience [26-30]. The current study also revealed that the participants in the

intervention arm tended to be more knowledgeable than their counterparts in the control arm, explaining the highly significant variation after intervention.

Previous studies ascertained that the perception and knowledge of breast cancer and prevention preferences increased after intervention [30]. This study also found that there was a strong interplay between perception and breast self-examination after intervention, demonstrating that participants in the intervention districts were more likely to consider them susceptible. This result is consistent with the previously documented studies [30-32]. This is congruent with the assumption of HBM that perceived susceptibility is better than real susceptibility to facilitate preventive health behaviors [17, 23]. In this study, the severity of the breast cancer was found to be the best predictor of the likelihood of screening behavior after intervention, producing a highly significant mean difference in the intervention group. The result is consistent with the study conducted in Addis Ababa and other countries [16, 33].

Previous studies documented that beliefs concerning the cause of the disease are a crucial determinant of subsequent screening [16; 34]. The current study also found that perceived benefits (provided there are no barriers) of screening methods had a significant effect on providing a preferred positive course of action. Surprisingly, this study found that the intervention group unexpectedly had higher barrier scores than the control group. Previous research outputs documented contradicting findings [29, 35]. The current study also found that intervention had significantly increased women's self-efficacy in breast screening. This is similar to the study conducted in Iran that showed total stigma and discrimination against cancer can be avoided through tailored education [29]. Similarly, self-efficacy is a predicting variable in several breast cancer studies and other studies too [28, 35].

Previously documented studies in Ethiopia had shown the influence of the media was very poor for a variety of economic, psycho-social, and cultural reasons [16, 36]. Likewise, the current study found that the influence of cues to action after intervention explained the least variance in the program.

In real science, health education is the best predictor of behavioral change [23]. The current study also found that practice of actual breast self-examination increased after intervention, where no cost is required. Previously published studies also supported this idea [28, 30].

This study found that health motivations were increased in the intervention arm. Previous publications also confirmed that successful motivational interventions confirm the persuasiveness of personal and individualized risks [13-15].

As strength of the study, this is one of the first randomized controlled trials in Ethiopia to implement community-based interventions beyond conventional observational studies like cross-sectional studies. The other strength of the study is that the research team used qualitative methods and other relevant reviews before designing and developing the intervention. This might help the researchers recognize the value of interventions and explore new intervention mechanisms with an accurate method rather than a mere description.

As limitations of the study, the study used health promotion interventions at the community level in case information contamination may exist due to the nature of health promotion research. The intervention document was prepared for reproductive-aged women, assuming the WHO guidelines for breast self-examination but not an officially approved protocol. The exact composition and peak of health education might not be known, or insufficiency may occur in

some cases. The benefits obtained from the educational intervention in the intervention group are an ethical question for not having a control group.

In conclusion, the findings of this study evidence the possibility that increasing community awareness and perceptions (beliefs) about the use of existing services increase the likelihood of healthy behavior. This study also indicated that community-based intervention followed by an exploratory qualitative approach through gap analysis had a significant impact on the likelihood of performing breast self-examination. To this end, the study pushed us to conclude that knowledge, perception, and future intentions of the women to prevent breast cancer are the important determinants of breast examination and that intervention should be sustained. As for the future prospects of the research, though RCT is the gold standard design for determining cause-and-effect relationships in effectiveness studies, various research approaches are necessary in order to evaluate the effectiveness of the dynamic nature of health promotion research. In our quest to understand breast self-examination better, further research is needed to assess the doze and the peak of intervention, where the actual behavior change is reached, to provide a complete picture of women‘s conceptualizations of breast self-examination.

Authors Contribution

FDA, GG, RS, ES, MY and ZB were involved in the design, conduct and analysis of the study. FDA prepared the manuscript for publication and all authors critically reviewed the manuscript.

Acknowledgments

First and foremost, we would like to express our gratitude to the International Campus of Tehran University of Medical Sciences for the scholarship grant, as well as for the correct review, and approval of this thesis work. We are also grateful to Wachemo University for providing financial

assistance and duplicate questionnaires. We would also want to thank the study participants for their substantial contributions to the research process.

Funding

Not applicable.

Consent for publication

Not applicable.

Competing interests

No conflict of interest to declare.

References

1. Puvanesarajah S, Gapstur SM, Patel AV, Sherman ME, Flanders WD, Gansler T, Troester MA, Gaudet MM. Mode of detection and breast cancer mortality by follow-up time and tumor characteristics among screened women in Cancer Prevention Study-II. Breast Cancer Research and Treatment. 2019 Oct;177:679-89.
2. Bertuccio P, Turati F, Carioli G, Rodriguez T, La Vecchia C, Malvezzi M, Negri E. Global trends and predictions in hepatocellular carcinoma mortality. Journal of hepatology. 2017 Aug 1;67(2):302-9.
3. Getu MA, Abebe M, Tlaye KG, Goshu AT. Breast self-examination knowledge and its determinants among female students at Addis Ababa University, Ethiopia: an institution-based cross-sectional study. BioMed Research International. 2022 May 30;2022.
4. Shita A, Yalew AW, Seife E, Afework T, Tesfaw A, Gufue ZH, Rabe F, Taylor L, Kantelhardt EJ, Getachew S. Survival and predictors of breast cancer mortality in South Ethiopia: A retrospective cohort study. Plos one. 2023 Mar 6;18(3):e0282746.
5. De Ver Dye T, Bogale S, Hobden C, Tilahun Y, Hechter V, Deressa T, Bize M, Reeler A. A mixed-method assessment of beliefs and practice around breast cancer in Ethiopia: implications for public health programming and cancer control. Global public health. 2011 Oct 1;6(7):719-31.
6. Tolessa L, Sendo EG, Dinegde NG, Desalew A. Risk factors associated with breast cancer among women in Addis Ababa, Ethiopia: unmatched case–control study. International Journal of Women's Health. 2021 Jan 18:101-10.

7. Parise CA, Caggiano V. Regional variation in disparities in breast cancer specific mortality due to race/ethnicity, socioeconomic status, and urbanization. Journal of racial and ethnic health disparities. 2017 Aug;4:706-17.
8. Tigeneh W, Molla A, Abreha A, Assefa M. Pattern of cancer in Tikur Anbessa specialized hospital oncology center in Ethiopia from 1998 to 2010. Int J Cancer Res Mol Mech. 2015 May 22;1(1):1.
9. Ayele W, Führer A, Braun GA, Formazin F, Wienke A, Taylor L, Unverzagt S, Addissie A, Kantelhardt EJ. Breast cancer morbidity and mortality in rural Ethiopia: data from 788 verbal autopsies. BMC Women's Health. 2022 Dec;22(1):1-9.
10. Kantelhardt EJ, Zerche P, Mathewos A, Trocchi P, Addissie A, Aynalem A, Wondemagegnehu T, Ersumo T, Reeler A, Yonas B, Tinsae M. Breast cancer survival in Ethiopia: a cohort study of 1,070 women. International journal of cancer. 2014 Aug 1;135(3):702-9.
11. Wubie A, Seid O, Eshetie S, Dagne S, Menber Y, Wasihun Y, Petrucka P. Determinants of chronic energy deficiency among non-pregnant and non-lactating women of reproductive age in rural Kebeles of Dera District, North West Ethiopia, 2019: Unmatched case control study. PloS one. 2020 Oct 29;15(10):e0241341.
12. Dinegde NG, Demie TG, Diriba AB. Knowledge and practice of breast self-examination among young women in tertiary education in Addis Ababa, Ethiopia. Breast Cancer: Targets and Therapy. 2020 Nov 3:201-10.
13. Mekonnen BD. Breast self-examination practice and associated factors among female healthcare workers in Ethiopia: a systematic review and meta-analysis. PloS one. 2020 Nov 10;15(11):e0241961.

14. Agide FD, Sadeghi R, Garmaroudi G, Tigabu BM. A systematic review of health promotion interventions to increase breast cancer screening uptake: from the last 12 years. European journal of public health. 2018 Dec 1;28(6):1149-55.
15. Agide FD, Garmaroudi G, Sadeghi R, Shakibazadeh E, Yaseri M, Koricha ZB, Tigabu BM. A systematic review of the effectiveness of health education interventions to increase cervical cancer screening uptake. European journal of public health. 2018 Dec 1;28(6):1156-62.
16. Getu MA, Abebe M, Tlaye KG, Goshu AT. Breast self-examination knowledge and its determinants among female students at Addis Ababa University, Ethiopia: an institution-based cross-sectional study. BioMed Research International. 2022 May 30;2022.
17. Glanz K, Rimer BK, Viswanath K, editors. Health behavior and health education: theory, research, and practice. John Wiley & Sons; 2008 Aug 28.
18. Hausmann-Muela S, Ribera JM, Nyamongo I. Health-seeking behaviour and the health system response. Disease Control Piroirities Project working paper No14. 2003
19. Agide FD, Garmaroudi G, Sadeghi R, Shakibazadeh E, Yaseri M, Koricha ZB. Likelihood of breast screening uptake among reproductive-aged women in Ethiopia: a baseline survey for randomized controlled trial. Ethiopian Journal of Health Sciences. 2019;29(5).
20. Mekuria M, Nigusse A, Tadele A. Breast self-examination practice and associated factors among secondary school female teachers in gammo gofa zone, southern, Ethiopia. Breast Cancer: Targets and Therapy. 2020 Jan 29:1-0.
21. World Health Organization. Training for mid-level managers. 2008
22. Teferi S, Mezgebe T, Demissie M, Durgaprasada A. Knowledge about breast cancer risk-factors, breast screening method and practice of breast screening among female healthcare

professionals working in governmental hospitals, Addis Ababa, Ethiopia. IOSR J Pharm Biol Sci. 2012;2(1):5-12.

23. Glanz, Karen Ed, Barbara K. Rimer, and K. Viswanath. "Theory, research, and practice in health behavior." (2015).

24. Snyman LC. Breast cancer mammography screening for low-risk women in South Africa. Southern African Journal of Gynaecological Oncology. 2010 Jan 1;2(2):69-70.

25. Akuoko CP, Armah E, Sarpong T, Quansah DY, Amankwaa I, Boateng D. Barriers to early presentation and diagnosis of breast cancer among African women living in sub-Saharan Africa. PloS one. 2017 Feb 13;12(2):e0171024.

26. Akhtar SS, Nadrah HM, Al Habdan MA, El Gabbani SA, El Farouk GM, Abdelgadir MH, Al Saigul AM. First organized screening mammography programme in Saudi Arabia: preliminary analysis of pilot round. EMHJ-Eastern Mediterranean Health Journal, 16 (10), 1025-1031, 2010. 2010.

27. Arshad S, Williams KP, Mabiso A, Dey S, Soliman AS. Evaluating the knowledge of breast cancer screening and prevention among Arab-American women in Michigan. Journal of Cancer Education. 2011 Mar;26:135-8.

28. Dallo FJ, Zakar T, Borrell LN, Fakhouri M, Jamil H. Cancer knowledge increases after a brief intervention among Arab Americans in Michigan. Journal of Cancer Education. 2011 Mar;26:139-46.

29. Taymoori P, Molina Y, Roshani D. Effects of a randomized controlled trial to increase repeat mammography screening in Iranian women. Cancer nursing. 2015 Jul;38 (4):288.

30. Tuzcu A, Bahar Z, Gözüm S. Effects of interventions based on health behavior models on breast cancer screening behaviors of migrant women in Turkey. Cancer nursing. 2016 Mar 1;39(2):E40-50.

31. Birhane N, Mamo A, Girma E, Asfaw S. Predictors of breast self-examination among female teachers in Ethiopia using health belief model. Archives of Public Health. 2015 Dec;73:1-7.

32. Farmanfarma KK, Kiasara SH, Hassanipour S, Salehiniya H. Non-Hodgkin's lymphoma in the world: an epidemiological review. WCRJ. 2020;7(6):e1520.

33. Meiser B, Quinn VF, Mitchell G, Tucker K, Watts KJ, Rahman B, Peate M, Saunders C, Geelhoed E, Gleeson M, Barlow-Stewart K. Psychological outcomes and surgical decisions after genetic testing in women newly diagnosed with breast cancer with and without a family history. European Journal of Human Genetics. 2018 Jul;26 (7):972-83.

34. Salisu WJ, Mirlashari J, Seylani K, Varaei S, Thorne S. Fatalism, distrust, and breast cancer treatment refusal in Ghana. Canadian Oncology Nursing Journal. 2022;32(2):198.

35. Eskandari-Torbaghan A, Kalan-Farmanfarma K, Ansari-Moghaddam A, Zarei Z. Improving breast cancer preventive behavior among female medical staff: the use of educational intervention based on health belief model. The Malaysian journal of medical sciences: MJMS. 2014 Sep;21(5):44.

36. Mekonen M. Assessment of Breast Self-Examination Practice and Associated Factors among Women between 20 up to 70 Years Attending on the Selected Public Health Centers in Addis Ababa, Ethiopia, 2015 (Doctoral dissertation, Addis Ababa University).

Figure
Click here to access/download;Figure;Figure 1 Consort flow Diagram.tiff

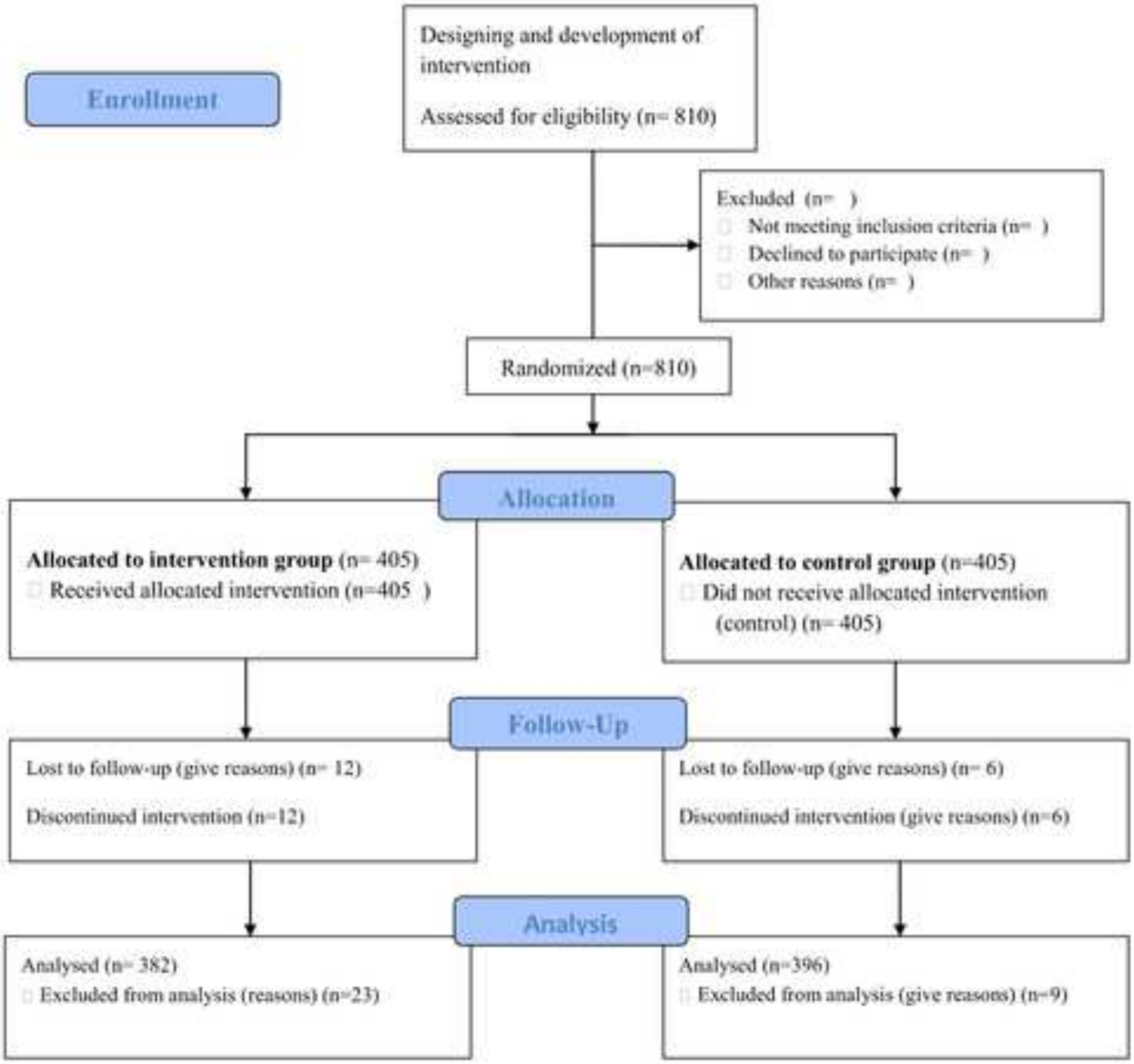

Figure 1: CONSORT flow diagram of the progress through the phases of a parallel randomized trial of two groups (that is, enrolment, intervention allocation, follow-up, and data analysis). Available at: http://www.consort-statement.org/consort-statement/flow-diagram

Figure

Click here to access/download;Figure;Figure 2.tiff

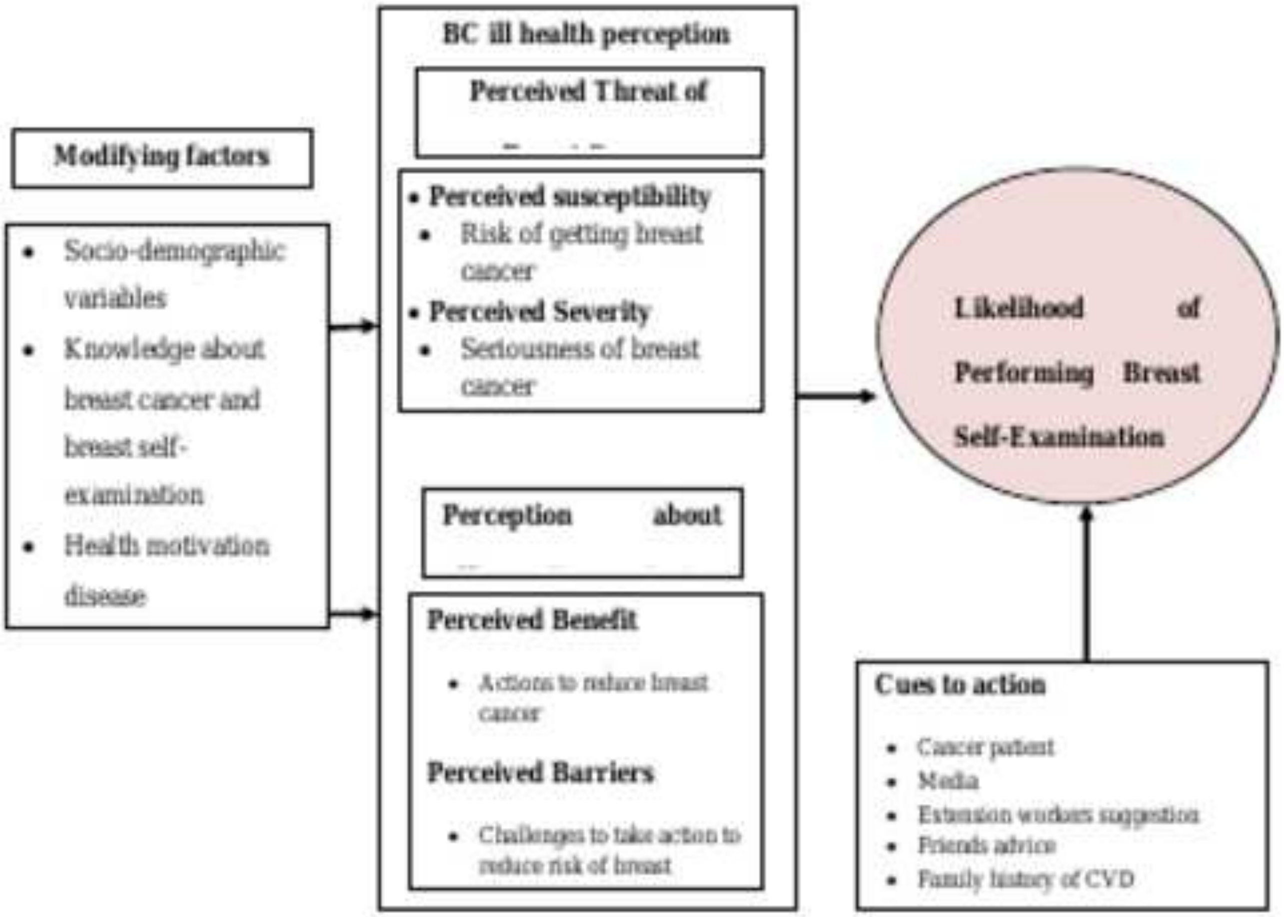

Figure 2: Conceptual framework of the research [17, 23].

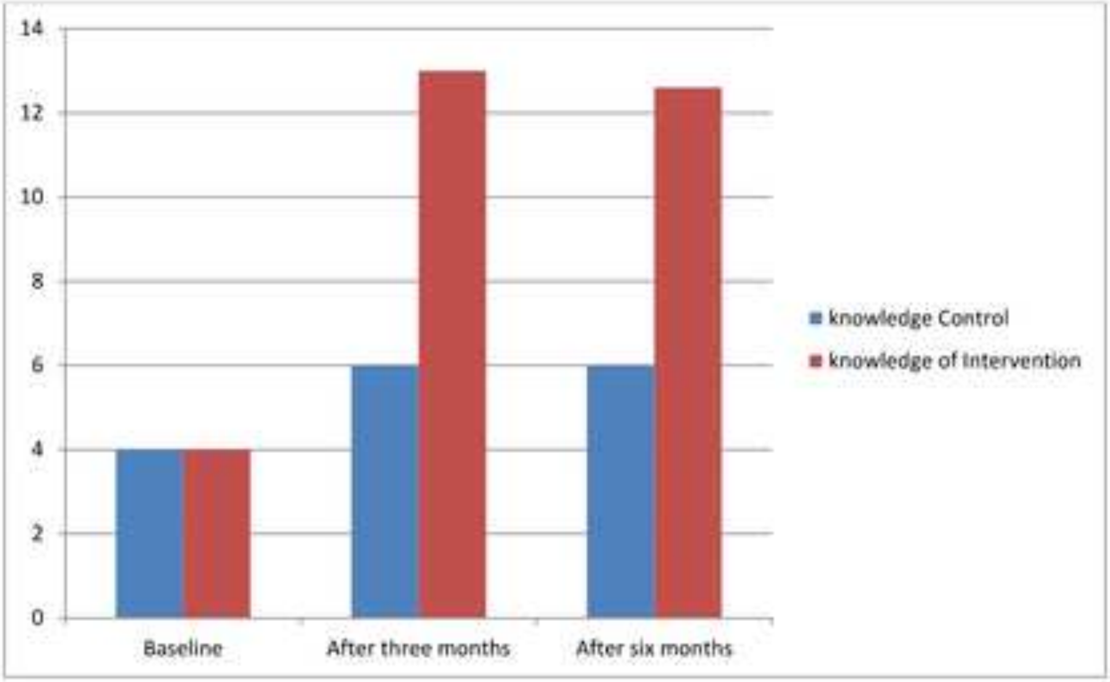

Figure 3: Knowledge of the participants about breast cancer and breast self-examination in intervention and control groups across the study period.

Click here to access/download
Supporting Information
CONSORT-2010-Checklist for RCT.doc

Click here to access/download
Supporting Information
Initial protocol.docx

Click here to access/download
Other
Ethics tums.jpg

Click here to access/download
Other
Ethics letter.jpg

Click here to access/download
Other
Ethiopian ethics.jpg
