# Supplementary material for: Designing and evaluation of the effect of community-based intervention on breast self-examination among reproductive-aged women in Ethiopia: A Cluster Randomized Controlled Trial
Source: PLoS One. 2025 Aug 7;20(8):e0329839. doi: 10.1371/journal.pone.0329839 (PMC12331034; doi:10.1371/journal.pone.0329839)
Supplement: Attachment — Submitted filename: Response-to-editors-and-reviewers- plose one.docx [file pone.0329839.s006.docx]

**Response to Editors and reviewers,**

We are happy to get the opportunity to revise our manuscript, titled **"Designing and evaluation of the effect of community-based intervention on breast self-examination among reproductive-aged women in Ethiopia: A Cluster Randomized Trial." Manuscript number: PONE-D-23-37776.** We appreciate your helpful comments, proposing an edit for further improvement, and your interest in the publication. We revised the editor's and reviewers' comments. The **"Revised Manuscript"** will show how much we worked on our manuscript based on your comments. The comments are valuable in improving the paper's quality. As a result, I, as the corresponding author, put the response to your comments **point-by-point**.

**Responses to Editor**

Dear respected editor, thank you for your valuable comments and interest in our manuscript for publication. Your comments were incorporated into the revised manuscript one-by-one.

**Comment 1:** How was the risk of unintended knowledge transfer between the intervention and control groups addressed, considering that both groups interacted with the same health extension workers?

**Authors Response:** Thank you for the valuable comment and professional view. We used the health extension workers from different (not included) woreda to deliver special prepared health education or intervention for intervention group; whereas, we use the existing health extension workers for the control group for usual education. We revised the manuscript in the way that your comment is being addressed.

**Comment 2:** What steps were taken to minimize this exposure, ensuring that the control group received no content related to the intervention beyond what was intended?

**Authors Response:** Thank you for the valuable professional view and comment. Since this is a health promotion intervention, we selected the intervention and control woreda from very distant places. All the intervention districts’ included kebele were categorized as intervention groups, and those control kebele were the control groups. Random assignment is made for kebele rather than individuals. Interventionists do not know for what purpose they are doing rather than recognizing the importance of intervention. We also revised this part in a way that your concern is being addressed.

**Comment 3:** What specific measures were implemented to ensure the integrity of the intervention was maintained?

Authors Response: Thank you for the valuable suggestion/comment. We prepared intervention protocol for the intervention delivering. i.e. **Clear definition of the intervention:** The intervention should be precisely defined, specifying the goals, components, target population, delivery methods, duration, and expected outcomes. A well-articulated protocol is essential for consistency across implementation. **Evidence-based framework:** The intervention should be grounded in a theoretical framework or an evidence-based approach that supports its feasibility and potential effectiveness in addressing the health issue based on qualitative data. Pre- and Post-Assessment: Conduct assessments before and after the intervention to measure changes in health behaviours. We supervised and trained to be always be truthful with participants, admit when they make a mistake, and treat everyone fairly. Show respect to participants and listen to their ideas. Stick to the rules and be a good role model in delivering specially prepared health education.

**Comment 4:** Reframe conclusions to align with the study’s specific scope.

**Authors Response:** Thank you for the comment**.** We reframed the conclusion again as per your comment to align with the study’s specific scope.

**Comment 5:** Ensure findings are directly tied to data collected within the intervention context, not external studies.

**Authors Response:** Thank you for the valuable suggestion**.** We revised your concern in the revised manuscript.

**Comment 6:** Proofread for grammatical errors, typos, and punctuation mistakes to improve clarity.

**Authors Response:** Thank you for the valuable suggestion/comment. We have tried to address the grammatical and punctuation issues in the revised manuscript.

**Comment 7:** Clearly state the Data Availability Statement in line with PLOS ONE’s Data Policy.

**Authors Response:** Thank you for the valuable comment. We revised Data Availability Statement accordingly.

**Comment 8:** Address all reviewer comments thoroughly, providing clear justifications.

**Authors Response:** Thank you for the valuable suggestion. We addressed all the comments of reviewers.

**Responses to Reviewer #1**

Thank you for your valuable comments and interest in our manuscript for publication. Your comments were incorporated into the revised manuscript and responded one by one.

**General comment:** A two-phase randomized trial was conducted which aimed to design and evaluate the effect of community-based intervention on breast self-exam among women in Ethiopia. The likelihood of both performing breast self-examination and the comprehensive knowledge increased from baseline to the end of the intervention period. Several other conclusions are unclear.

**Authors Response:** Thank you for your valuable comment. We reframed the conclusion again as per your comment to align with the study’s specific scope.

**Comment 1:** Data collection, quality management, processing, and analysis section: The chi-square and ANOVA tests are not appropriate for comparing rates of breast self-examination at baseline, after three months and at the end of six months since an underlying assumption of these tests is independent samples.

**Authors Response:** Thank you for your valuable comment. We addressed your comment in the revised manuscript.

**Comment 2:** Table 5: In the title indicate if these results are from univariate or multivariate models. They appear to be univariate models, meaning that only one variable at a time has been included in the model. Consider fitting a multivariate model.

**Authors Response:** Thank you for your valuable comment. We addressed your comment in the revised manuscript. We have also tried to clearly state how the model is fitted in the analysis part. Our analysis were done according to your comment.

**Comment 3:** Thoroughly explain the data presented in Table 7.

**Authors Response:** Thank you for your valuable comment. Path analysis was done to determine the direct and indirect effects of constructs of health belief model (HBM) and to estimate the values of coefficients in the underpinning linear model. Path analysis is simply standardized partial regression coefficient partitioning the correlation coefficients into measures of direct and indirect effects of a set of independent variables on the dependent variable. We revised your concern in the revised manuscript.

**Comment 4:** In the statistical analysis section, identify the statistical testing methods used to estimate the p-values in Table 1.

**Authors Response:** Thank you for your valuable comment. We used simply Pearson chi-square. We deleted it since it has no use.
**Comment 5:** P-values never equal zero; express small p-values as < 0.001.

**Authors Response:** Thank you for your valuable comment. We revised your concern in the revised manuscript.
**Comment 6:** Express p-values more precisely than “p< 0.05.”

**Authors Response:** Thank you for your valuable comment. We revised your concern in the revised manuscript.
**Comment 7:** To assist in the review process, add line numbering to the document.

**Authors Response:** Thank you for your valuable comment. We added the line number in the revised manuscript.

**Responses to Reviewer #2**

Thank you for your valuable comments and interest in our manuscript for publication. Your comments were incorporated into the revised manuscript and clarified one by one.

**General comment of Reviewer #2:** I enjoyed reading this insightful and informative submission and consider it a significant contribution to the literature on women's health. However please address some minor comments to enhance the quality of the manuscript.

**Authors Response:** Thank you for your valuable interest and comments.
**Comment 1** Abstract: Method section: It is better to write the study design method. This is a mixed-method study.
**Authors Response:** Thank you for your valuable comment. We revised your concern in the revised manuscript.
**Comment 2:** It is better to write briefly the eligibility criteria for participants, interventions intended for each group, how participants were allocated to interventions, tools used, time, and the settings where the data were collected.

**Authors Response:** Thank you for your valuable comment. We revised your concern in the revised manuscript.
**Comment 3:** Findings: State baseline characteristics at the beginning of the findings.
**Authors Response:** Thank you for your valuable comment. We revised your concern in the revised manuscript.
**Comment 4:** Introduction: The introduction was well written.

Authors Response: Thank you for your encouraging comment.
**Comment 5:** Method: What do you mean by the following phrase for Exclusion criteria?” people who could move during the intervention,”

**Authors Response:** Thank you for your valuable comment. We want to say individuals who will not stay until the intervention is completed/until the last data collection time for some reasons. That means those individuals who left the environment for some reason before three times of data collection were excluded.

**Comment 6:** Given that the qualitative results have been published elsewhere, it would have been better if you had not mentioned the two-phase study design and had only described the second phase.

**Authors Response:** Thank you for your valuable comment. We revised your concern in the revised manuscript. Basically, our intention is to show that our intervention is data driven and gap recognized through qualitative method. It is a sequential exploratory design.
**Comment 7:** How was the validity and reliability of the constructed instrument achieved?

**Authors Response:** Thank you for your comment. We have conducted the Cronbanch’s alpha for reliability analysis. We have conducted pre-test in five present of the study population and the necessary adjustments were made in the questionnaire part. All this is there in the revised manuscript.
**Comment 8:** Considering that the intervention content is prepared from the qualitative phase, it is better to also include a description of the content of the sessions.

**Authors Response:** Thank you for your valuable comment. We described a description of the content of the sessions in the revised manuscript.

**Comment 9:** The phrase "oral informed consent" is repeated twice in one paragraph.

**Authors Response:** Thank you for your valuable comment. We edited in the revised manuscript.

**Comment 10:** It is better to separate the data analysis from the data collection in the method section. It would be better to delete the following sentence:” then analyzed and interpreted by the research team and a biostatistician”

**Authors Response:** Thank you for your valuable comment. We deleted and revised your concern in the revised manuscript. We also separated data analysis from the data collection in the method section.
**Comment 11:** Do you have any potential confounders? How to control confounding effects by statistical analysis.

**Authors Response:** Thank you for your valuable comment. We did regression analysis to avoid potential confounding effects and we revised your concern in the revised manuscript.
**Comment 12:** Which test was used to test the normality of the distribution of the response variables? Please write the significance level in the analysis section.
**Authors Response:** Thank you for your valuable comment. The Kolmogorov-Smirnov test was used to test the null hypothesis that a set of data comes from a normal distribution (P>0.05). We revised your concern in the revised manuscript.

**Comment 13:** Findings: The figures are not clear. (Words are unreadable.)

**Authors Response:** Thank you for your valuable comment. We revised your concern in the revised manuscript.
**Comment 14:** In all tables, 0.000 should be corrected as follows <0.001

**Authors Response:** Thank you for your valuable comment. We revised your concern in the revised manuscript.
**Comment 15:** Please indicate the type of statistical tests used at the bottom of the tables.

**Authors Response:** Thank you for your valuable comment. We revised your concern in the revised manuscript.

**Comment 16**: What is meant by the two interventions in the following sentence?” there was no significant difference between the two interventions (6±1.75 and 6±1.78)”

**Authors Response:** Thank you for your valuable comment. We edited the typos error in the revised manuscript. In the control group, the mean (4.0±2.11) showed a slight increase after three months and at the end of the intervention, whereas there was no significant difference between the two times of data collection in control group (6±1.75 and 6±1.78).

**Comment 17:** How was the effect of the initial significant difference in educational and occupational status between the intervention and control groups on the outcome controlled

**Authors Response:** Thank you for your valuable comment. Educational status is the level of education whereas occupational status is the working condition.

**Comment 18:** Explain how missing data were addressed. Please state the reasons for excluding from analysis.

**Authors Response:** Thank you for your valuable comment. To address missing data, common strategies include deletion (removing cases with missing values). We also used imputation (replacing missing values with estimates).

**Comment 20:** Discussion: Considering the large volume of findings, they are not well addressed in the discussion section.

**Authors Response:** Thank you for your valuable comment. We enriched our document and wrote the discussion part again.

**Comment 21:** Interpretations of the results and the consistency and inconsistency of the findings with other studies need to be improved.

**Authors Response:** Thank you for your valuable comment. We improved your concern in the revised manuscript.

**Responses to Reviewer #3**

Thank you for your valuable comments and interest in our manuscript for publication. Your comments strengthen and improved our manuscript. Thank you again. Your comments were incorporated into the revised manuscript and clarified one by one.

**General Comment of Reviewer #3**: The manuscript's intention to see the effect of community based educational intervention on improving BSE practice is superb. However, the conclusion is made not only from this particular manuscript but other proceeding and related thesis works. Therefore, the conclusion should be modified based on this interventional study's findings. The authors used different statistical techniques to prove their point, but the manuscript lacks clarity on the measurement of outcome variable(s). The methods section should elaborate why they prefer each method of analysis in detail. The design itself is not clear from the title whether it is cluster randomized trial or clinical trial. The randomization process differs for clusters and individuals, so it should be clear and it seems a mixture of both techniques from the manuscript. There are few areas that needs correction on grammar, punctuation and typos. The data availability is not explicitly stated in the mother document.

**Authors Response:** Thank you for your positive comment and professional view. We thoroughly revised your concern and the data availability is explicitly stated in the revised manuscript. **General Comment of Reviewer #3**: The comments and suggestions are given on the pdf for detail consideration, clarification and correction. Please, see the attached pdf.

**Authors Response:** Thank you for your valuable comment. We addressed comments and suggestions are given on the pdf in the revised manuscript.

**Thank you again for thoroughly reviewing our manuscript!!**
