# Supplementary material for: Designing and evaluation of the effect of community-based intervention on breast self-examination among reproductive-aged women in Ethiopia: A Cluster Randomized Controlled Trial
Source: PLoS One. 2025 Aug 7;20(8):e0329839. doi: 10.1371/journal.pone.0329839 (PMC12331034; doi:10.1371/journal.pone.0329839)
Supplement: Attachment — Submitted filename: Response-to-editors and reviewers minor revision.docx [file pone.0329839.s007.docx]

**Response to Editors and reviewers,**

We are happy to get the opportunity to revise our manuscript, titled **"Designing and evaluation of the effect of community-based intervention on breast self-examination among reproductive-aged women in Ethiopia: A Cluster Randomized Controlled Trial" Manuscript number: PONE-D-23-37776.** We appreciate your helpful comments, proposing an edit for further improvement, and your interest in the publication. We revised the reviewer's comments. As a result, I, as the corresponding author, put the response to your comments **point-by-point**.

**Responses to Editor**

**General Comments of editor:** - Please complete a thorough proofread of the text and correct any spelling and grammar errors that you identify. We encourage you to revise the manuscript with careful consideration of all reviewer feedback. Indicate which changes you require for acceptance versus which changes you recommend. Address any conflicts between the reviews so that it's clear which advice the authors should follow. Provide specific feedback from your evaluation of the manuscript

**Authors Response:** Thank you for the valuable comment. We revised all your concerns in the revised manuscript and edited as well. We would like to inform you that we have benefited from your revision and the manuscript is improved well.

**Responses to Reviewer #1**

Thank you for your valuable comments and interest in our manuscript for publication. Your comments were incorporated into the revised manuscript and responded one by one.

**General comment:** The standard statistical term for average is mean.

**Authors Response:** Thank you for your valuable comment. We used mean as a statistical standard term in the revised manuscript.

**General comment:** Table 3: Express p-values more precisely than "p<0.05".

**Authors Response:** Thank you for your valuable comment. We expressed p-values more precisely in the revised manuscript.

**Responses to Reviewer #2**

Thank you for your valuable comments and interest in our manuscript for publication. Your comments were incorporated into the revised manuscript and clarified one by one.

**General comment of Reviewer #2:** All Comments have been addressed in the revised manuscript. Please implement these 3 comments to improve the manuscript. Abbreviations should not be written in the abstract (HBM), In all tables, 0.000 should be corrected as follows <0.001. You did not write this sign [<]. Please indicate the type of statistical tests used at the bottom of the tables.

**Authors Response:** Thank you for your valuable interest and comments. The HBM is now written in full form. We also corrected 0.000 as the <0.001 in all tables. We also indicated the type of statistical tests used at the bottom of the tables.

**Responses to Reviewer #3**

**General comment of Reviewer #3**: This version of the manuscript is more improved except few typos and grammatical errors. I hope the authors go over it one more time before publication.

**Authors Response:** Thank you for your valuable comments and interest. We edited for grammar and typos as much as possible.

**Thank you again for thoroughly reviewing our manuscript!!**
