# Supplementary material for: Designing and evaluation of the effect of community-based intervention on breast self-examination among reproductive-aged women in Ethiopia: A Cluster Randomized Controlled Trial
Source: PLoS One. 2025 Aug 7;20(8):e0329839. doi: 10.1371/journal.pone.0329839 (PMC12331034; doi:10.1371/journal.pone.0329839)
Supplement: Attachment — Submitted filename: 2 Response-to-editors minor revision.docx [file pone.0329839.s008.docx]

**Response to Editors and reviewers,**

We are pleased to get the opportunity to revise our manuscript, titled **"Designing and evaluation of the effect of community-based intervention on breast self-examination among reproductive-aged women in Ethiopia: A Cluster Randomized Controlled Trial" Manuscript number: PONE-D-23-37776.** We value your interest in the publication, your insightful comments, and your suggestion for an edit to make it even better. We updated the reviewers' and editor's comments. The comments are really helpful in raising the quality of the work. Since we rectified every concern brought up, we think the paper's quality has much improved. I will thus respond to each of your comments point-by-point as the corresponding author.

**Responses to Editor**

**General Comments of editor:** Ensure all reviewer comments have been fully addressed. Edit for grammar, spelling, and punctuation throughout. The category “Others” in Table 2 under “Frequency of breast screening” should be clearly defined. Please specify what behaviours or responses are included in “Others.” Perform a final proofreading for flow, transitions, and logical structure.

**Authors Response:** Thank you for the valuable and professional comments. We edited for grammar, spelling, punctuation, structure and flow throughout the manuscript. We specified the category “Others” and revised all your concerns in the revised manuscript and edited as well. We would like to inform you that we have benefited from your revision and the manuscript is improved well.

**Responses to Reviewer #1**

Thank you for your valuable comments and interest in our manuscript for publication. Your comments were incorporated into the revised manuscript and responded one by one.

**General comment:** 1- Table 6: For p-values between 1.0 and 0.001, replace the less than sign with an equals sign.

**Authors Response:** Thank you for your valuable comment. We accepted your comment and we replaced the less than sign with an equals sign in the revised manuscript. In addition to that we grammar, spelling, punctuation, structure and flow throughout the manuscript.

**Thank you again for thoroughly reviewing our manuscript!!**
