# Supplementary material for: Designing and evaluation of the effect of community-based intervention on breast self-examination among reproductive-aged women in Ethiopia: A Cluster Randomized Controlled Trial
Source: PLoS One. 2025 Aug 7;20(8):e0329839. doi: 10.1371/journal.pone.0329839 (PMC12331034; doi:10.1371/journal.pone.0329839)
Supplement: Attachment — Submitted filename: 3 Response-to-editors minor revision.docx [file pone.0329839.s009.docx]

**Response to Editors and reviewers,**

We are pleased to get the opportunity to revise our manuscript, titled **"Designing and evaluation of the effect of community-based intervention on breast self-examination among reproductive-aged women in Ethiopia: A Cluster Randomized Controlled Trial" Manuscript number: PONE-D-23-37776.** We appreciate your helpful comments, proposing an edit for further improvement, and your interest in the publication. We revised the reviewer's comments. As a result, I, as the corresponding author, put the response to your comments **point-by-point.**

**Responses to Editor**

**General Comments of editor:** Please review your reference list to ensure that it is complete and correct. If you have cited papers that have been retracted, please include the rationale for doing so in the manuscript text, or remove these references and replace them with relevant current references. Any changes to the reference list should be mentioned in the rebuttal letter that accompanies your revised manuscript. If you need to cite a retracted article, indicate the article’s retracted status in the References list and also include a citation and full reference for the retraction notice.

**Authors Response:** Thank you for the concern and professional comments. We checked references whether retracted or not.

**Responses to Reviewer #1**

Thank you for your valuable comments and interest in our manuscript for publication. Your comments were incorporated into the revised manuscript and responded one by one.

**Comment:** **1-** For results where p > 0.05, please report the exact p-values to two significant digits. Additionally, for non-significant p-values, remove the less-than sign. For example, in Table 3, the p-value for Health Motivation is currently reported as '< 0.135'; this should be revised to '0.135'. Similar inconsistencies appear throughout the document and should be addressed accordingly.

**Authors Response:** Thank you for your valuable comment. We accepted your comment and we put the exact p-values as per your comment in the revised manuscript.

**Comment: 2-** In Table 5, change the column header from "Sig." to "p-value" for clarity and consistency.

**Authors Response:** Thank you for your valuable comment. We accepted your comment and we changed the column header from "Sig." to "p-value" in the revised manuscript.

**Comment: 3-** When reporting p-values, always use a lowercase p. There is currently a mix of uppercase and lowercase Ps in Table 6 that should be standardized.

**Authors Response:** Thank you for your valuable comment. We accepted your comment and we used a lowercase p in the revised manuscript.

**Thank you again for thoroughly reviewing our manuscript!!**
