# Supplementary material for: Designing and evaluation of the effect of community-based intervention on breast self-examination among reproductive-aged women in Ethiopia: A Cluster Randomized Controlled Trial
Source: PLoS One. 2025 Aug 7;20(8):e0329839. doi: 10.1371/journal.pone.0329839 (PMC12331034; doi:10.1371/journal.pone.0329839)
Supplement: Attachment — Submitted filename: Response to Reviewers.docx [file pone.0329839.s010.docx]

**Response to Editors and reviewers,**

We are happy to get the opportunity to revise our manuscript, titled **"Designing and evaluation of the effect of community-based intervention on breast self-examination among reproductive-aged women in Ethiopia: A Cluster Randomized Controlled Trial" Manuscript number: PONE-D-23-37776.** We appreciate your helpful comments, proposing an edit for further improvement, and your interest in the publication. We revised the reviewer's comments. As a result, I, as the corresponding author, put the response to your comments **point-by-point**.

**Responses to Editor**

**General Comments of editor:** Please include the following items when submitting your revised manuscript:

- A rebuttal letter that responds to each point raised by the academic editor and reviewer(s). You should upload this letter as a separate file labelled 'Response to Reviewers'.
- A marked-up copy of your manuscript that highlights changes made to the original version. You should upload this as a separate file labelled 'Revised Manuscript with Track Changes'.
- An unmarked version of your revised paper without tracked changes. You should upload this as a separate file labelled 'Manuscript'.

**Authors Response:** Thank you for the valuable comment. We prepared and submitted 'Response to Reviewers', 'Revised Manuscript with Track Changes' and 'Unmarked version Manuscript'. We would like to inform you that we have benefited from your revision and the manuscript is improved well.

**Responses to Reviewer #1**

Thank you for your valuable comments and interest in our manuscript for publication. Your comments were incorporated into the revised manuscript and responded as well.

**General comment:** When reporting p-values that are less than 0.0001, please include the ‘<’ symbol (i.e., report as p < 0.0001). This is a standard convention to ensure precision and clarity in statistical reporting.

**Authors Response:** Thank you for your valuable comments and interest. We added the **‘<’** symbol before <0.0001 in all tables. I also consulted a senior statistician, he advised me to use less than sign in both cases (P<0.001 and <0.0001).

**Thank you again for thoroughly reviewing our manuscript!!**
